# Metapangenomics reveals depth-dependent shifts in metabolic potential for the ubiquitous marine bacterial SAR324 lineage

**DOI:** 10.1186/s40168-021-01119-5

**Published:** 2021-08-13

**Authors:** Dominique Boeuf, John M. Eppley, Daniel R. Mende, Rex R. Malmstrom, Tanja Woyke, Edward F. DeLong

**Affiliations:** 1 Daniel K. Inouye Center for Microbial Oceanography: Research and Education, University of Hawaii, Manoa, Honolulu, HI 96822 USA; 2 DOE Joint Genome Institute, Berkeley, CA 94720 USA

**Keywords:** Marine microbiome, Microbial ecology, Ecotype, Pangenomic, Metagenomic, Metatransciptomic, Plankton, Deep ocean, Photoheterotrophy, Chemoautotrophy

## Abstract

**Background:**

Oceanic microbiomes play a pivotal role in the global carbon cycle and are central to the transformation and recycling of carbon and energy in the ocean’s interior. SAR324 is a ubiquitous but poorly understood uncultivated clade of Deltaproteobacteria that inhabits the entire water column, from ocean surface waters to its deep interior. Although some progress has been made in elucidating potential metabolic traits of SAR324 in the dark ocean, very little is known about the ecology and the metabolic capabilities of this group in the euphotic and twilight zones. To investigate the comparative genomics, ecology, and physiological potential of the SAR324 clade, we examined the distribution and variability of key genomic features and metabolic pathways in this group from surface waters to the abyss in the North Pacific Subtropical Gyre, one of the largest biomes on Earth.

**Results:**

We leveraged a pangenomic ecological approach, combining spatio-temporally resolved single-amplified genome, metagenomic, and metatranscriptomic datasets. The data revealed substantial genomic diversity throughout the SAR324 clade, with distinct depth and temporal distributions that clearly differentiated ecotypes. Phylogenomic subclade delineation, environmental distributions, genomic feature similarities, and metabolic capacities revealed strong congruence. The four SAR324 ecotypes delineated in this study revealed striking divergence from one another with respect to their habitat-specific metabolic potentials. The ecotypes living in the dark or twilight oceans shared genomic features and metabolic capabilities consistent with a sulfur-based chemolithoautotrophic lifestyle. In contrast, those inhabiting the sunlit ocean displayed higher plasticity energy-related metabolic pathways, supporting a presumptive photoheterotrophic lifestyle. In epipelagic SAR324 ecotypes, we observed the presence of two types of proton-pumping rhodopsins, as well as genomic, transcriptomic, and ecological evidence for active photoheterotrophy, based on xanthorhodopsin-like light-harvesting proteins.

**Conclusions:**

Combining pangenomic and both metagenomic and metatranscriptomic profiling revealed a striking divergence in the vertical distribution, genomic composition, metabolic potential, and predicted lifestyle strategies of geographically co-located members of the SAR324 bacterial clade. The results highlight the utility of metapangenomic approaches employed across environmental gradients, to decipher the properties and variation in function and ecological traits of specific phylogenetic clades within complex microbiomes.

Video abstract

**Supplementary Information:**

The online version contains supplementary material available at 10.1186/s40168-021-01119-5.

## Background

Oceanic microbiomes provide a pivotal ecosystem service by buffering the rise of atmospheric CO_2_ through carbon sequestration in the ocean’s interior mainly via the biological carbon pump [1]. The demand in organic carbon by heterotrophic microbiota from the dark ocean, the Earth’s main remineralizers, exceeds the production by phytoplankton from the sunlit ocean [2]. Chemoautotrophic activity in the dark ocean has been suggested to be on a similar order of magnitude as heterotrophic activity and thus might contribute considerably to carbon and energy recycling in the ocean’s interior [3]. Because the dark ocean comprises about 75% of the global ocean’s volume and contains 98% of the global dissolved inorganic carbon (DIC) pool [4], microbes that fix inorganic carbon are critical components for deep-sea carbon cycling.

Since its first identification in the Sargasso Sea [5], the SAR324 clade, a monophyletic group of marine Deltaproteobacteria recently proposed to be reclassified into its own phylum [6], has been recognized as globally relevant in the ocean aphotic zone [7–10]. Remarkably, SAR324 SSU rRNA gene variants are present throughout the water column from surface waters to the abyss [5, 11], with abundance maxima correlating with low-oxygen concentration [5]. In the oxygen minimum zone (OMZ) notably, especially in dysoxic and suboxic waters, SAR324 make up a significant component of the microbial community [5, 12]. Despite their ubiquity and abundance, no SAR324 representatives have been cultivated to date. Our understanding of their metabolism and ecological role therefore rests on few genomic fragments [7, 11, 13], environmental genomes reconstructed from metagenomes and metatranscriptomes [14, 15], and single-cell amplified genomes (SAGs) [16, 17]. Available genomes of SAR324 have been predominantly used to explore metabolic capabilities of the mesopelagic zone inhabitants [17], or those found near deep hydrothermal plumes [14, 15]. Common metabolic traits reported for SAR324 representatives include high plasticity in metabolic features, the presence of genes required to fix inorganic carbon and to metabolize C1 compounds, and the ability to oxidize sulfur. These data suggest the potential for a chemoautotrophic lifestyle of SAR324 representatives that live in the dark ocean. Although considerable, progress made in elucidating the metabolic potential of the SAR324 clade has been limited to the dark ocean. Less is known about the ecology and metabolic potential of this clade in the euphotic and twilight zones of the upper water column.

To better define the nature of the SAR324 pangenome [18, 19] throughout the open ocean water column, we conducted an integrated multi-omics study from the surface waters to the abyss at Station ALOHA (22.75° N, 158° W: A Long-term Oligotrophic Habitat Assessment), in the North Pacific Subtropical Gyre (NPSG). In the NPSG, the permanently stratified surface layer restricts the vertical fluxes of inorganic nutrients from deeper waters and results in a broad range of habitats from warm, light-saturated, nutrient-starved surface waters to the cold, dark, high-pressure, nutrient-rich abyss [20–22]. In this study, we built a locally relevant pangenome from SAR324 single-cell amplified genomes (SAGs) sampled throughout the water column from surface to the abyss (− 4000 m), to compare, analyze, and explore the genomic features and metabolic capabilities of this ubiquitous group. Genomic profiling of SAR324 SAGs, along with time-resolved metagenomic depth profiles [23] and high-frequency Lagrangian metagenomic and metatranscriptomic diel surveys [24], provided the environmental and biological context to assess the ecology of SAR324 populations, and revealed the spatio-temporal variability of their key metabolic potentialities.

## Main text

### Results

#### SAR324 population genome binning

We generated 14 deltaproteobacterial SAGs from seven depths at Station ALOHA and retrieved additional draft SAR324 genomes (28 metagenomic assembled genome (MAGs) and 6 SAGs, [17, 25–31]) from public databases, screened for similarity to Station ALOHA populations by metagenomic read mapping. Since MAGs intrinsically represent a possible consensus of several genomes within a population, and SAGs are incomplete and fragmented, we designed an approach delimiting operational sets of genetically related genomes to analyze functions of a population rather than a single genome. Average nucleotide identity (ANI) was used to assess the relationship between the set of selected draft genomes, and a threshold of 95% was set as a boundary for population delineation [32, 33]. Genes from the genomes within a population were cataloged (i.e., clustered by similarity and dereplicated) and treated as a “population genome”. From this, a dataset of 48 draft SAGs and MAGs (completeness average, 54.1%; min, 4.2–max, 91.3) comprising 98,323 predicted genes was reduced to 18 population genomes (completeness average, 62.4%; min, 17.4–max, 96.6), yielding 51,089 predicted genes. Both the low completeness of genomes representative of natural populations and the low recovery of the flexible genome by MAGs could lead to misinterpreting of core and plastic functions among pangenome. This approach reduced sample complexity by decreasing the functional redundancy (and removing non-coding space), simultaneously improving the completeness of population representative genome, from a gene-centric perspective (Supp. Table 1). In addition, mixing functional genes from both MAGs and SAGs into operational bins allowed to reduce uncertainties inherent to both techniques of genome generation.

#### ANI clustering and classification of SAR324 genomes

**Fig. 1:**
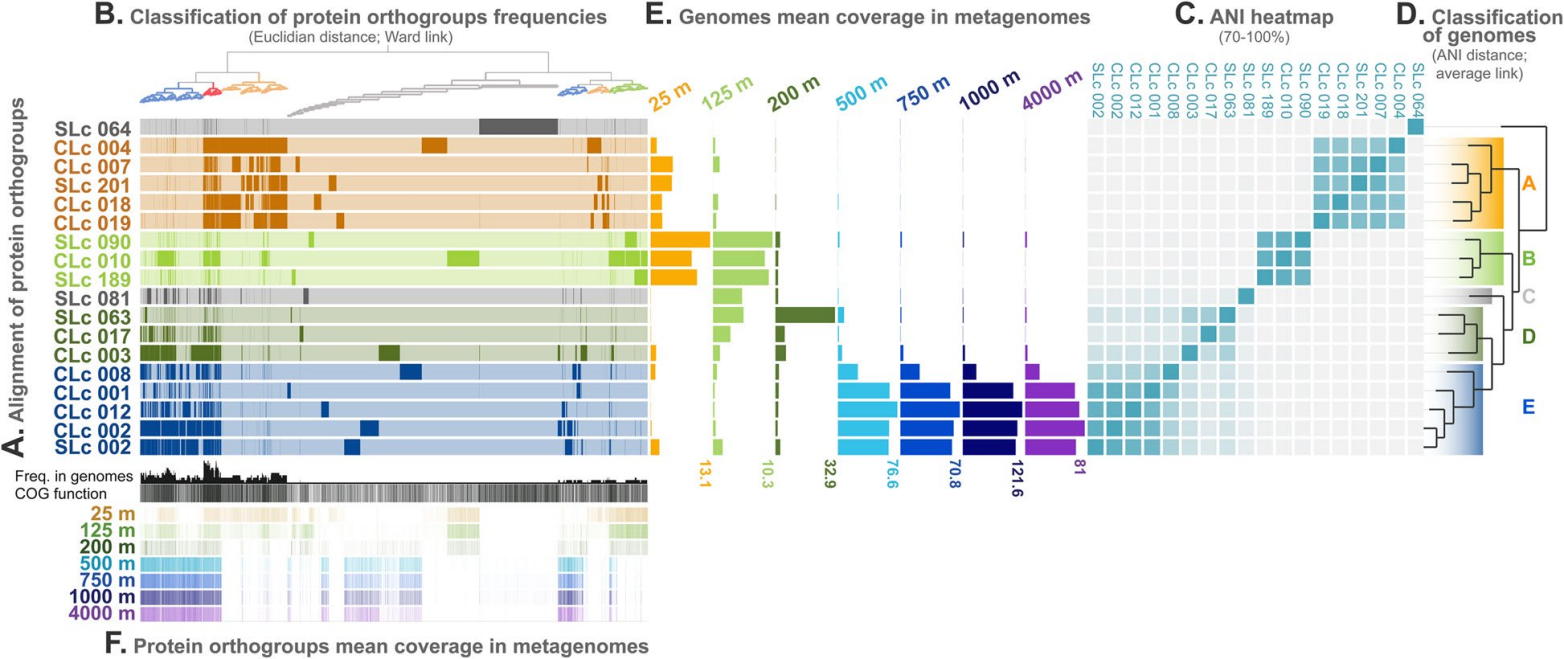
Local metapangenome of SAR324 at Station ALOHA. **A** Pangenome. Alignment of protein orthogroups among population genomes constituting the pangenome. Population genomes without a significant amount of metagenomic reads mapped (**E**) have been removed to keep only locally relevant genomes. Colored bars indicate the presence of a protein into the population genome. Population genomes have been colored according to subclade/ecotype definition as suggested by ANI classification (**D**) and depth distribution (**E**). **B** Ordering of protein orthogroups by Ward classification of Euclidean distances between frequencies of orthogroups in SAR324 pangenome. Branches are colored according to frequencies: orthogroups from core genome (genes present in all population genomes) in red; orthogroups common to subclade A in yellow, subclade B in green, and subclades C, D, E in blue; and singletons (protein unique to a population genome) are in gray. **C** Square heatmap of average nucleotide identity between population genomes ordered according to ANI classification of population genomes (**D**). Scaled from 70 to 100% of identity. **D** Hierarchical ascendant classification based on the average link of ANI distance between populations genomes. **E** Average coverage of population genomes by metagenomic reads from depth of SAGs sampling across the water column at Station ALOHA. **F** Average coverage of protein orthogroups by metagenomic reads from depth of SAGs sampling across the water column at Station ALOHA

Using ANI as a distance metric (Fig. 1C, D), the 18 population genomes were classified into one NB1-j outgroup (SLc_064) and five subclades that could be affiliated to SAR324 (sensu Wright et al. [5]) as identified by their 16S rRNA genes (Supp. Figure 4). These comprised: five population genomes (CLc_004, CLc_007, SLc_201, CLc_018, CLc_019) grouped into subclade A, three population genomes (SLc_090, CLc_010, SLc_189) into subclade B, a single SAG from this study (SLc_081) composed the subclade C, three population genomes (SLc_063, CLc_017, CLc_003) grouped into subclade D, and five population genomes (CLc_008, CLc_001, CLc_012, CLc_002, SLc_002) into subclade E. Genomic GC percentages (Supp. Table 1) were different between outliers (66.6%) and SAR324 genomes (43.03% ± 3 (SD)). GC content was conserved within subclades, supporting their close phylogenetic relationships: subclade A (45.64% ± 1.21), subclade B (46.59% ± 1.28), subclade D (41.98% ± 2.08), and subclade E (41.53% ± 0.83). Genomic GC percentages were slightly different between the population genomes (containing only coding sequences) and individual genomes that composed them (1.01 ± 0.02%), supporting the congruence of genomic features between the genomes pooled together into population genomes.

Subclade definition by ANI clustering (with more than 80% ANI between subclades) was congruent with the one obtained using classical phylogenetic marker genes like the 16S rRNA gene (Supp. Figure 5). Placing the 16S rRNA genes from our SAR324 genomes by parsimony into the SILVA reference phylogenetic tree (Supp. Figure 4), produced clusters similar, but less resolutive, to those based on ANI of genomes. Using ANI distance to address phylogenetic relationships allowed us to finely cluster partial genomic fragments even if they did not harbor a satisfying set of phylogenetic markers (16S rRNA gene [34] or universal single-copy marker genes [35]).

#### SAR324 abundance in metagenomes and ecotypes delineation

To assess environmental representativity of SAGs, metagenomes were constructed from the samples that SAGs were isolated from in 2015/2016. Metagenomic reads were mapped onto the SAR324 genes extracted from population genomes. Average coverages over the different population genomes were used to assess their depth distributions (Fig. 1E). Distributions of the population genomes were depth-specific, with internal subclade consistency, supporting a phylogenetically coherent ecotype distribution. Subclade A was only present in surface waters but in lower abundance than the subclade B that was distributed above the DCM (maximal between 75 and 125 m but not present below 200 m deep). Subclade C and D were present at the DCM and immediately below (125–200 m). Subclade E was abundant below 500 m (500, 750, 1000, 4000 m) reaching the highest abundance for the entire clade at 1000 m deep (almost tenfold higher than surface abundance). Hence, 4 ecotypes were defined, based on genomic similarity and depth distribution: surface ecotype (subclade A), above-DCM ecotype (subclade B), below-DCM ecotype (subclades C and D), and deep ecotype (subclade E).

#### The metapangenome of SAR324

Predicted proteins from population genomes were aligned and clustered into groups of proteins dubbed hereafter as orthogroups (groups of orthologous proteins) representing the pangenome of SAR324 (Fig. 1A). Orthogroups were classified and displayed according to their frequencies in population genomes (Fig. 1B). A small number of orthogroups (225 groups, see Venn diagram in Supp. Figure 1 A) were shared among all the SAR324 genomes analyzed (i.e., “core genome”, in red, Fig. 1B). A higher proportion of orthogroups was shared exclusively among subclades (“core” genome of subclade A in yellow, subclade B in green, subclades D and E in blue, Fig. 1B). A total of 2674 orthogroups among all 4533 specific orthogroups were shared between all genomes within the subclade A (Supp. Figure 1 A), while 1064 among 2530 were shared within the subclade B, 159 orthogroups were shared among the 1079 for the subclade D, and 994 among 3392 total for the subclade E.

As displayed by high ANI values, genomes from subclades D and E were closely related, sharing many orthogroups in common (976 orthogroups exclusively shared between subclades D and E). More shared orthogroups (612) were obtained when genomes were grouped by ecotypes (Supp. Figure 1 B) rather than by subclades. Surface ecotypes and deep ecotypes encompassed most of the unique orthogroups (4533 and 3392, respectively). The highest number of shared orthogroups (1303) was found between the below-DCM and deep ecotypes.

The SAR324 core genome was distributed evenly across all depths, while accessory genome showed depth-specific distributions (Fig. 1F). Although the bias induced by the incompleteness of genomes used to build the pangenome cannot be waived, grouping population genomes allowed to display trends in orthogroups scattering among ecotypes. Core genome accounted for about 12% (9.5 to 14.5%) of the orthogroups of each ecotype (Supp Table 3) while singletons (i.e., orthogroups found in a single population genome only) accounted for about 35% (28.5 to 39.1%). About half of the total amount of orthogroups (53%) was shared between genomes within a single ecotype or into at least two ecotypes. Interestingly, orthogroups unique to a given ecotype were in a higher proportion at surface (44%) than in deeper layers (1.7 to 13.8%).

#### Time and depth distribution of SAR324 ecotypes at Station ALOHA in 2011

**Fig. 2:**
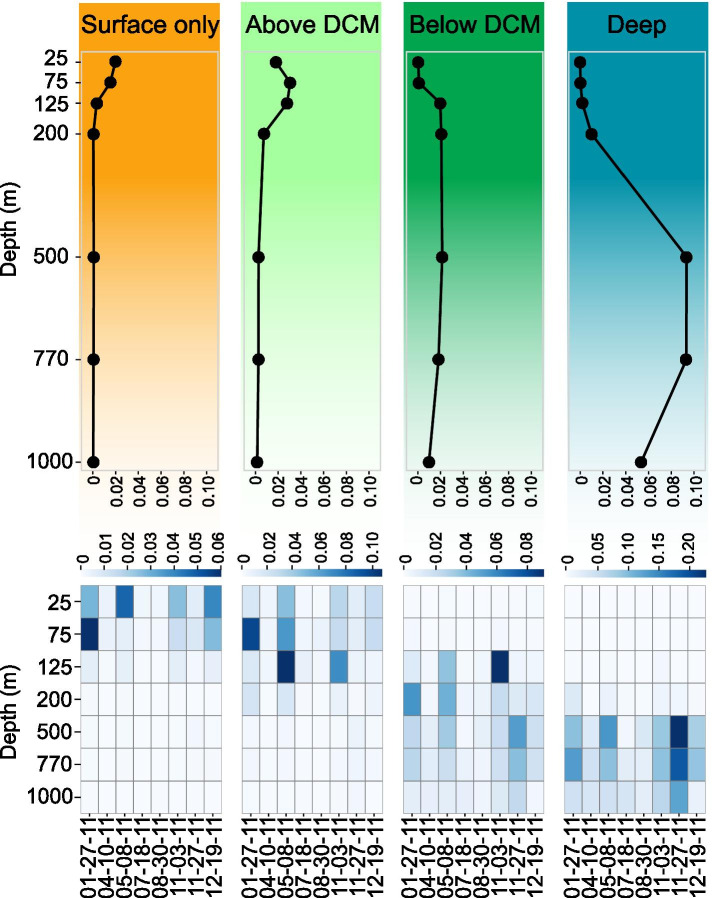
Distribution of SAR324 ecotypes at Station ALOHA. **A** Average distribution of SAR324 ecotypes according to depth. SAR324 population genomes coverage by metagenomic reads from HOT time-series have been averaged by ecotypes, by depth and by date over the time-series. **B** Average distribution of SAR324 ecotypes according to time. SAR324 population genomes coverage by metagenomic reads from HOT time-series have been averaged by ecotypes and by depth date over the time-series

To determine SAR324 ecotype depth distributions across time, average coverages were assessed by mapping them with metagenomic time-series reads from Station ALOHA (Fig. 2). The general depth distributions averaged across all dates (Fig. 2A) displayed the clear depth zonation of ecotypes. Depth distributions (Fig. 2B) showed consistent patterns of ecotype zonation with time, but with divergent temporal variations in abundance for each ecotype. Surface ecotypes were more abundant at 25 m and 75 m, with a deeper distribution in January and December. Above-DCM ecotypes had higher abundance at 125 m in May and early November, and high abundance at 75 m in January. The below-DCM ecotype had a high abundance at 125 m in November but was consistently present at 200 m and deeper. The deep ecotype was mostly restricted to water masses below 500 m, with higher abundances at the end of November at 500 m and 750 m.

The deep chlorophyll maximum (DCM) zone (fluctuating around 125 m deep) and the nutricline in the disphotic zone (steep change in nutrients concentration and water masses characteristics between 200 and 500 m deep) appeared to be two biological relevant boundaries for the ecotype distribution of SAR324. Environmental parameters fluctuated more over time in the euphotic zone (Supp. Figure 2), and the depth of the DCM varied slightly with time at Station ALOHA which could explain the higher variability in time and depth of distribution maxima for shallower ecotypes compared to those of the deeper ones.

#### Metabolic pathway reconstruction and distribution

**Fig. 3:**
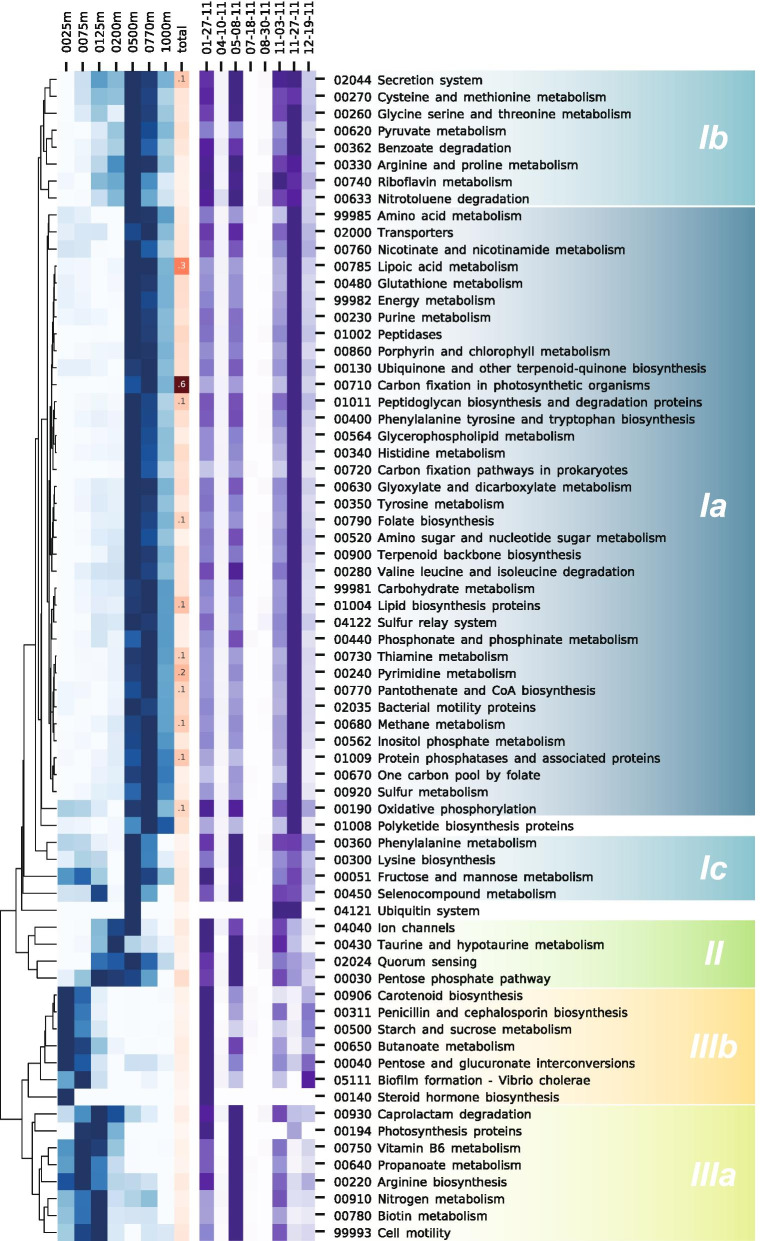
Distribution of SAR324 metabolic pathways at Station ALOHA. Hierarchically clustered heatmap of depth (blue) distribution of metabolic pathways in metagenomes from HOT time-series. Heatmap of distribution over time has been overlaid in purple. Depth and time distribution of each pathway have been scaled from 0 to 1. Average coverage over depth is displayed in red for each pathway. Color backgrounds have been applied to pathway clusters according to the depth at which their relative abundance peaked, from yellow in the surface to dark blue in the deep

To reconstruct metabolic pathways of each ecotype, protein orthogroups from the pangenome were annotated and explored using the KEGG database. Coverage within each relevant metabolic pathway across depth and time was averaged and clustered according to depth profile (Fig. 3). Metabolic pathways grouped into distinguishable clusters with defined depth and temporal distributions. Like ecotype depth distributions, the DCM and nutricline coincided with the boundaries separating metabolic pathway distributions. Most pathways were abundant at 500 m and 750 m (cluster Ia) with diverse pathways involved in carbon fixation, utilization of C1 (THF) and C2 (Glyoxylate) compounds, phosphonates, sulfonates, and sulfur. All those pathways followed the same temporal patterns with a maximal abundance in November. A group of pathways (cluster Ib), comprising degradation of nitrotoluene and benzoate, displayed tight distribution patterns with higher abundance from 125 to 1000 m and a maximum at 500 m. Cluster Ib was abundant when the abundance of SAR324 members peaked, in January, May, and early and late November. Cluster Ic, including fructose, mannose, and selenocompound metabolic pathways, had less defined distributions and were absent at 200 m depth. The pentose phosphate pathway, taurine metabolism, and ion transporters were grouped into cluster II that reached a maximum around 200 m, with highest abundance in January and May. Clusters IIIa and b were distributed in surface waters with maxima at 75–125 m and 25 m, respectively. The cluster IIIb was mainly present in January and IIIa in May. Remarkably, carotenoid biosynthesis pathway, starch and sucrose metabolism, and butanoate metabolism were present only at 25 m (IIIb). Nitrogen metabolism and vitamins metabolisms (B6 and B8), as well as structures involved in cell motility, were mostly found above 200 m (cluster IIIa).

#### Key enzyme and protein distributions

**Fig. 4:**
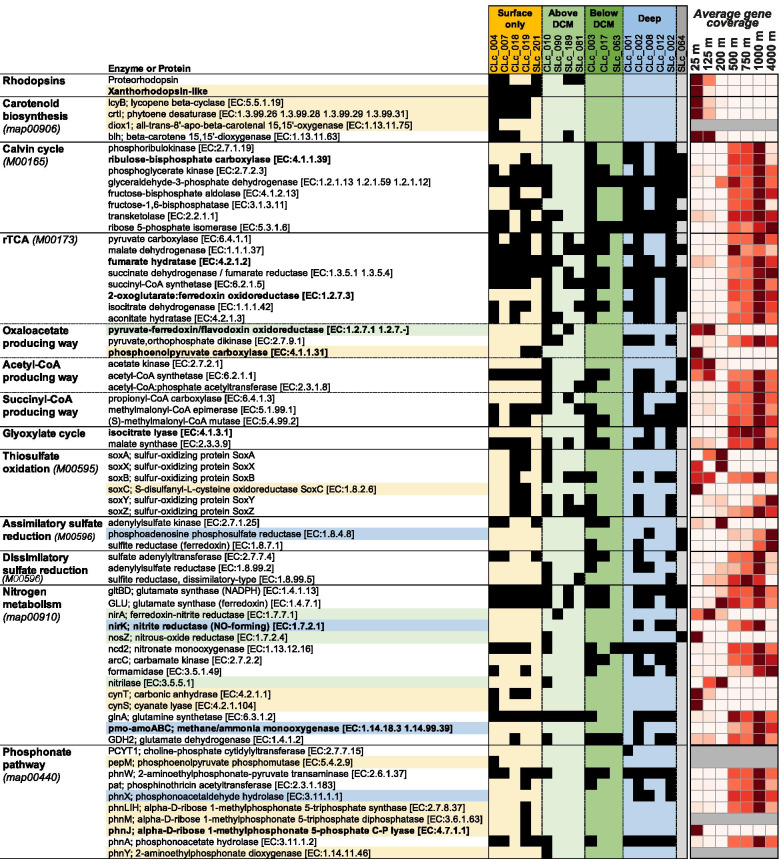
Distribution of SAR324 key enzymes in population genomes and in metagenomes at Station ALOHA. Presence of genes coding for key enzymes in population genomes are displayed in black. Enzymes that are present in a single subclade are colored by their subclade. Depth distribution of proteins is displayed in red shade; distributions are scaled from 0 (white) to 1 (red); proteins without metagenomic coverage are in dark gray

The presence of enzymes or proteins involved in key reactions of the metabolism (central carbon metabolism, source of electron, nutrients use) was identified among population genomes and ecotypes by KEGG gene annotations (Fig. 4). To verify that gene distributions reflected genome distributions (and were not artificially due to genome incompleteness), the coverage for each gene in metagenomes associated with SAGs was computed and scaled from 0 to 1 to smooth the effect of gene copies (right panel in Fig. 4). The presence and distribution of key enzymes were structured according to depth, in a similar pattern as what was seen for metabolic pathways and ecotypes, with the DCM and nutricline corresponding also to metabolic boundaries. Central metabolic characteristics, including inferred sources of carbon and energy, coincided with the vertical zonation of ecotypes within the SAR324 clade.

#### Carbon metabolism

All SAR324 population genomes and ecotypes harbored the full tricarboxylic acid cycle (TCA) and pentose phosphate cycles, as well as complete glycolytic pathways (Supplementary maps ko00010, ko00020, ko00030). SAR324 genomes from all depths also encoded for C1-compounds use pathways, through the folate and tetrahydrofolate (THF) cycles (Supplementary maps ko00670, ko00790). In addition, the glyoxylate shunt of the TCA cycle, especially the key isocitrate lyase, was encoded in SAR324 genomes from ecotypes below the DCM and preferentially distributed in metagenomes below 200 m (Fig. 4).

SAR324 genomes from different ecotypes encoded different carbon fixation pathways. Genomes from below 200 m harbored the full Calvin-Benson-Basham (CBB) cycle, including the key enzyme ribulose-bisphosphate carboxylase (RuBisCO) (Fig. 4). Below-200 m genomes harbored type Ia RuBisCO, able to perform carboxylation reaction, while the two population genomes from above 200 m displayed type IV, a RuBisCO-like protein (RLP) involved in other reactions, possibly the biosynthesis of low-molecular-weight thiols essential for oxidation of thiosulfate and elemental sulfur (Supp. Figure 10). Type Ia RuBisCO from SAR324 showed active transcription in metatranscriptomes from 4000 m (data not shown [36]). The aerobic carbon monoxide dehydrogenase (CoxSLM), that oxidizes carbon monoxide to carbon dioxide under aerobic conditions, was present in almost all SAR324 population genomes analyzed, but was preferentially distributed below 200 m.

The fumarate hydratase and 2-oxoglutarate:ferredoxin oxidoreductase coding genes (Fig. 4, Supplementary map ko00720), allowing the TCA cycle to function in reverse (rTCA) and fix carbon, were present in genomes along with the canonical TCA enzymes (Supplementary map ko00020). Genes coding for enzymes involved in TCA cycle were mostly found in below-200 m metagenomes, while the fumarate hydratase gene was also detected in above-200 m metagenomes. Genomes from the surface ecotype possessed the gene coding for the phosphoenolpyruvate (PEP) carboxylase, which provides oxaloacetate to TCA/rTCA by fixing bicarbonate to PEP. The gene encoding the carbonic anhydrase, which converts carbon dioxide in bicarbonate used by PEP carboxylase was also only present above 200 m.

The complete C1-based tetrahydrofolic acid cycle (THF) was present in SAR324 genomes and distributed below 200 m (Supplementary map ko00670). In addition to the THF cycle, the presence in deep genomes and metagenomes of multiple genes could support a putative carbon dioxide fixation through methanogenesis (Supplementary map ko00680). Notable were present genes coding for the methane/ammonia monooxygenase (AmoA), for oxidoreductases involved in trimethylamine metabolism, such as the trimethylamine monooxygenase (Tmm) and the heterodisulfide reductase (HdrA) as well as the hydrogenase (HyaAB), and for the F420 co-enzyme biosynthesis pathway.

SAR324 genomes from divergent ecotypes harbored different ABC transporters (Supplementary map ko02010), including for potential carbon sources, with marked depth distribution (Supp Figure 3). For example, fructose and urea transporters were preferentially distributed above the DCM while glucose/mannose between 125 and 200 m and phospholipids and amino acids below 200 m.

#### Sulfur metabolism

Multiple enzymes involved in sulfur metabolism were encoded in SAR324 genomes (Fig. 4). Genes coding for SOX system (or TOMES, Thiosulfate Oxidation MultiEnzymes System), oxidizing thiosulfates to provide reducing power for cell, were present in population genomes from all ecotypes, but the system was not complete in any ecotype. The SoxYZ complex (sulfur chelating and binding proteins) pivotal proteins of TOMES that covalently bound sulfur substrate were found in all ecotypes. Sulfohydrolase SoxB, that hydrolyzes SoxY-cysteine-S-sulfate liberating sulfate, was present and distributed throughout the entire water column except for 200 m. At the 200-m boundary, only the heterodimeric cytochrome *c* SoxXA complex, that oxidatively couples the sulfane sulfur of thiosulfate to a SoxY-cysteine-sulfhydryl group of the SoxYZ complex, was detected.

In addition to SoxXA, SoxYZ, and SoxB, the gene coding for SoxC (sulfane dehydrogenase), forming the SoxCD complex, was present in SAR324 genomes from surface populations and detected into surface metagenomes (Fig. 4). This distribution of Sox complexes suggests that SAR324 performed thiosulfate oxidation through the canonical Kelly-Friedrich (Sox) pathway at the surface. A RuBisCO-like protein (RLP, type IV) that may be involved in biosynthesis of low-molecular-weight thiol essential for oxidation of thiosulfate and elemental sulfur, was encoded in one of the surface population genomes (CLc019) as well as in the outgroup SLc064 (Supp Figure 10).

SoxYZ complex and SoxB were the only part of the SOX system found in below-200 m genomes and metagenomes, respectively. However, genes coding for other enzymes related to sulfur utilization were found and preferentially distributed below 200 m such as those coding for the adenosine 5′-phosphosulfate (APS) reductase (AprAB), for the membrane QmoABC complex (Quinone-interacting membrane-bound oxidoreductase, the electron acceptor for AprAB), and for the ATP-sulfurylase (sat, a homolog of QmoABC generating ATP from APS). Genes for assimilatory sulfate reduction (notably ferredoxin-based and PAPS (CysH) reductases) were also detected in genomes below 200 m and distributed preferentially in deep metagenomes. Dissimilatory-type sulfite reductase (DsrAB) was present in both above 200 m and deeper populations. The heterohexameric DsrEFH and DsrC proteins, suspected to transfer sulfur from a persulfurated carrier molecule to the dissimilatory sulfite reductase DsrAB, were present in three population genomes (CLc010, SLc002, and SLc081) from above-DCM and deep ecotypes. The simultaneous presence of the SoxYZ-B complex, DsrAB, the DsrEFH-C complex, the AprAB-QmoABC complex, and ATP-sulfurylase (sat) below 200 m suggested that SAR324 from the deep are able to use the branched pathway for thiosulfate oxidation through reverse dissimilatory sulfate reduction (rDSR) and APS processing.

SAR324 genomes also harbored two ways to retrieve sulfite from the environment: from alkanesulfonate with the transporters (SsuACB) and the monooxygenase (SsuD) in genomes from above 200 m and from taurine with transporters (TauACB) and dioxygenase (TauD) in genomes from below 200 m.

The dimethylpropiothetin dethiomethylase (DddL), a carbon–sulfur lyase producing acrylate from DMSP (and producing DMS as byproduct), was present in genomes above 200 m. Acrylate may be converted into acryloyl-CoA by the propionyl-CoA synthetase (PrpE) then transformed into 3-hydroxypropionate-CoA by the enoyl-CoA hydratase (PaaF), feeding the TCA/rTCA cycle. In deeper populations, genes coding for the dimethylsulfide dehydrogenase (DdhABC) were found suggesting that DMS might also be oxidized into DMSO.

#### Nitrogen metabolism

Analyzed SAR324 genomes did not display nitrate/nitrite transporters nor enzymes to reduce, by any pathways, nitrate into nitrite. However, most of them possessed nitronate monooxygenase (Ncd2, NMO), oxidizing nitroalkane into nitrite and a formamidase, hydrolyzing formamide into formate and ammonium (Fig. 4). Above-200 m genomes harbored the ferredoxin-nitrite reductase (NirA) to reduce nitrite into ammonium as well as a nitrilase to hydrolyze nitrile into ammonium. No genomic evidence was found to support the use of nitrate to oxidize sulfur compounds.

Surface genomes possessed *cyn*S and *cyn*T genes coding for cyanate lyase and carbonic anhydrase, respectively. From cyanate and bicarbonate, cyanate lyase produces carbamate, that is spontaneously transformed into ammonium and carbon dioxide which is then recycled into bicarbonate.

Without associated pathways detected in this dataset, the *nos*Z gene coding for the nitrous oxide reductase was detected in one above-DCM genome, the nitric oxide-forming reductase (NirK) and the methane/ammonia monooxygenase (PmoA/AmoA) were present in some deep genomes.

Most analyzed genomes also coded for urease, degrading urea into ammonia and carbon dioxide, and its associated transporters. Considering all these findings, the ability to access and assimilate nitrogen contained in organic compounds (urea, nitroalkane, formamide, cyanate, possibly isocyanates, and related compounds) may be an important capability of the SAR324 lineage.

#### Phosphorus metabolism

Transporters for phosphates (PstABCS) were only detected in above-200 m genomes and were mostly distributed in metagenomes from above the DCM while transporters for phosphonates (PhnCDE) were present in most of genomes and preferentially distributed below 200 m. Most genomes possessed phosphonoacetate hydrolase (PhnA), catalyzing the hydrolytic cleavage of phosphonoacetate to inorganic phosphate (Fig. 4). Phosphonoacetaldehyde hydrolase (PhnX) was present and distributed in genomes and metagenomes from the deep. Interestingly, the key C-P lyase (PhnJ), that cleaves PRPn to retrieve phosphorus from organic compounds and release methane, was only present in surface genomes and only distributed in 25-m metagenomes.

#### Rhodopsin-based phototrophy

Two orthologous groups of rhodopsins were harbored by SAR324 genomes, both from above 200 m (Fig. 4). Protein sequence homology with close orthologs from Station ALOHA metagenomes (Supp Figure 7) and phylogenetic placement into a type II-rhodopsin reference tree (Supp Figure 6, [37]) revealed that one group was homologous to proteorhodopsin (PR) (environmental cluster 4, super-cluster I) and the other was distant from known rhodopsins and was most similar to xanthorhodopsins (XR). Proteorhodopsins are transmembrane, retinal-binding proteins that utilize light energy to transport protons outside the cell, generating a proton motive force across the membrane [38, 39]. All PR-like sequences from SAR324 population genomes had the DTE-Q motif of key amino acid residues in transmembrane helix 3, with a key lysine that binds retinal on transmembrane helix 7, suggesting that they function as blue-tuned proton pumps. XR-like SAR324 sequences also encoded the key lysine residue on the seventh helix and had a DTE-L motif in the third helix. XR-like type amino acid sequences were identical to a full length rhodopsin isolated from Station ALOHA metagenomes (HOT226_1_0075m_c26173_1) that, when expressed in *E. coli*, bound retinal and catalyzed light-driven proton pumping [40]. The leucine in position 105 suggests that XR-like rhodopsins are tuned to absorb in green light. Tertiary structure prediction from amino acid sequences confirmed the 7 transmembrane helix tunnel structure for both PR and XR-like types (Supp Figure 7).

PR and XR were both present in genomes of both surface ecotypes, but XR was absent in the above-DCM populations (Fig. 4). Gene distributions in metagenomes showed congruent patterns with PR type distributed in 25 m and 125 m while XR-like type only at 25 m. Enzymes (lycopene beta-cyclase, phytoene desaturase and beta-carotene oxygenase) involved in the biosynthesis of retinal were all present in surface genomes but absent from above-DCM ones. Genes encoding them were only distributed in surface metagenomes. Only one of the two oxygenases, the beta-carotene 15,15′-dioxygenase (Blh), performing the last step of β-carotene cleavage, was present in one of the above-DCM population genomes and distributed down to 125 m.

Synteny maps for XR-like (Supp Figure 8) and PR type (Supp Figure 9) opsins were evaluated by BlastN searches. Genes flanking PR type were not conserved among SAR324 genome variants, except between two genomes from subclade A. Only a contig from the SLc 189 population genome had a flanking gene involved into the retinal biosynthesis (*blh* gene coding for the beta-carotene 15,15′- dioxygenase). In contrast, genomic regions flanking XR-like encoding genes were highly conserved among all the SAR324 genomes. XR-like genes were directly adjacent to a complete set of genes encoding enzymes for retinal biosynthesis.

**Fig. 5:**
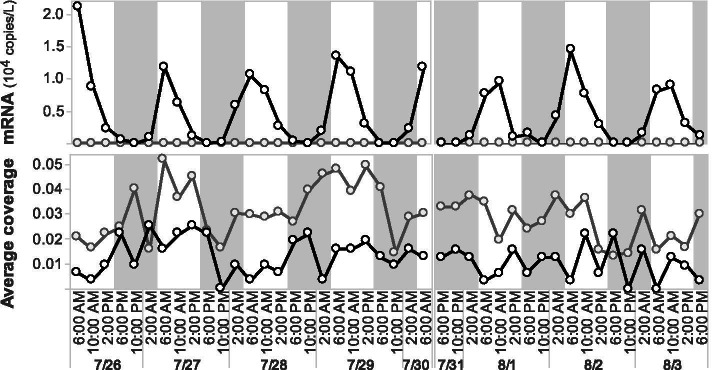
Diel average coverage (bottom) and transcription (top) of xanthorodopsin-like (black) and proteorhodopsin (gray) proteins in metagenomes and metatranscriptomes from HOE-legacy cruise II time-series (2016). Gray shades displayed night times

Distributions in time of PR and XR-like types were explored by metagenomic read mapping, which revealed little temporal variability among the different ecotypes bearing them (data not shown). Diel variability in SAR324 opsin gene abundance and transcription was explored via metatranscriptomic and metagenomic read mapping (Fig. 5). PR gene transcription was below the limit of detection, while XR-like opsins exhibited diel transcript abundances, with a XR transcript maxima occurring early in the morning each day.

### Discussion

Detailed investigation of distribution and dynamics of key planktonic microbial groups is required to better understand their adaptation to physico-chemical and biotic gradients encountered across the ocean’s vertical dimension but also to assess their depth-specific role in global biogeochemical cycles. The two most notable examples of vertically distributed, depth-adapted microbial groups in oligotrophic environments are the alphaproteobacterial SAR11 clade, the most abundant oceanic heterotroph, and *Prochlorococcus*, the most abundant oceanic oxygenic photoautotroph. Depth distributions include adaptive responses to temperature, light intensity, and nutrient availability for *Prochlorococcus* [41–43] and to seasonal, nutrient, and physico-chemical variation for SAR11 [44, 45]. Among other marine prokaryotes, less is known, especially for lineages, like SAR324, that inhabit the entire water column.

Consistent with previous reports based on SSU rRNA genes [5, 11], this study confirmed that SAR324 genomes were distributed from surface to abyssal zone at Station ALOHA. Representative SAR324 genomes were recovered throughout the entire water column, and specific populations organized to evaluate their genomic contents into a local pangenome. All the 48 draft genomes present locally at Station ALOHA were reduced to 18 population genomes, based on genome identity. The population genomes were clustered into 5 subclades according to their ANI distance. Using a depth resolved metagenomic time-series (7 discrete depths) we defined at least four ecotypes with specific depth and time distributions at Station ALOHA. This vertical zonation is likely the resultant of differential adaptations to nutrient, oxygen availability and hydrostatic pressure.

Previous studies based on internal transcribed spacers (ITS) of SSU rRNA coding gene [8] and SSU rRNA gene-containing fosmids [11] of SAR324 revealed that phylogenetic diversity exists within the SAR324 clade. It has been previously suggested [46] that ecotypes represent genetically closely related groups of bacteria, that are ecologically similar to one another (e.g., they occupy the same niche). Consistent with this concept, congruence between spatial and environmental partitioning, phylogenetic structure, and phenotype, have been demonstrated in *Prochlorococcus* [42]*.* However, such coupling between phylogenetic structure and spatio-temporal distribution are not as clear for other cosmopolitan groups like SAR11, with some genetically distant SAR11 subclades partitioning along similar geographic and seasonal distributions [45].

Reduction of dataset complexity is necessary to interpret ecologically relevant information from vast genomic complexity, especially nowadays with the large and growing number of available genomes. In this study, by binning closely related genomic contigs (i.e., genomes) as measured by their genetic features (ANI), and by cataloging non-redundant genes from each ANI-cluster of genomes (“population genome”), we reduced the complexity behind population representative genomes while keeping the functional information. This approach facilitated access to functional information from genomes within a population and eases the functional comparison between ecotypes within a clade. Metapangenomics, connecting pangenome to metagenomes, revealed to be a powerful technique to explore at a fine scale the genomic determinants underlying the ecotypes distribution of both *Prochlorococcus* and SAR11 [43, 47, 48]. Mapping metagenomic reads from depth and time-resolved environmental time-series on the locally relevant pangenome we built, allowed us to highlight strong congruences between notions of subclade (intra-clade diversity), genomic features similarity, ecological partitioning, and metabolic capabilities among SAR324 populations.

Based on genomic, metabolic, and ecologic similarities, four genomically consistent ecotypes of SAR324 were defined at Station ALOHA. Each ecotype displayed different depth and temporal distributions and divergent metabolic features. Two ecotypes inhabited the euphotic zone, with one found only in surface waters and another distributed from the surface to the DCM. Two other ecotypes dwelled deeper in twilight (ca 200–500 m) and dark oceans, respectively. Genomes of the two deeper ecotypes were more closely related, and their abundances were higher than SAR324 surface populations, with the ~ 200-m deep nutricline forming a strong boundary for SAR324 ecotype distributions. SAR324 ecotype abundances also distinctively varied in time, with their maximal abundances peaking at different periods during the year. Consistent with a previous study at Station ALOHA [49], all the SAR324 ecotypes displayed seasonal distributions with maximal abundances peaking during low-light periods of the year. Deeper ecotypes peaked at the end of the year and euphotic-inhabiting ecotypes peaked couple of months after, at the beginning of the year. Some previous reports suggest a particle-attached lifestyle for SAR324 [17, 50] but the strong discrepancy in gene contents between the SAR324 ecotypes delineated in this study, in addition to recent fine characterization of particle microbiota at Station ALOHA [36], did not support this hypothesis in this environment. Thus, the temporal shifting in distributions was unlikely due to the export of SAR324 populations from the euphotic zone to the deep ocean through sinking particles. However, the environmental parameters or biological interactions that drive SAR324 spatio-temporal distribution still remain to be elucidated, especially in the deep ocean which display very low variations in environmental parameters throughout the year.

Surprisingly, few genes were shared between all the SAR324 population genomes (core genome about 12% of orthogroups) but more among members within the same subclade or the same ecotype (Fig. 1A, B, Supp Figure 1). More genes were in common when genomes were grouped by ecotypes than when it was by genetic subclades, suggesting that SAR324 genomes share genes specific to the water layer they inhabited instead of an accessory genome in accordance with phylogenetic inertia. As suggested in a recent large-scale multi-pangenomic study, the heterogenous nature of aquatic habitats is suspected to be involved in the greater variability and the globally smaller core genomes of pangenomes recovered from those habitats, contrasting with those from soil, plants, and animal microbiomes [51]. The high proportion of the ecotype-specific genome regarding to the core genome in the SAR324 pangenome might indicate adaptation processes to the high variability in environmental conditions and the permanently stratified waters encountered throughout the water column at Station ALOHA. Indeed, metabolic pathways of the different SAR324 populations formed distinguishable clusters with specific depth and time distributions. SAR324 ecotypes inhabiting euphotic and aphotic layers exhibited the most dramatic shifts in metabolic capabilities and pathways. As suggested previously [14, 17], SAR324 may engage in chemoautotrophy in the dark ocean, utilizing *bona fide* RuBisCO, and the complete Calvin-Benson-Bassham (CBB) cycle to fix CO_2_. SAR324 ecotypes from aphotic zone appear to encode both the CBB and parts of the reverse TCA (rTCA) carbon-fixing pathways. Notwithstanding the presence of two of key enzymes of the rTCA cycle, namely the pyruvate and the 2-oxoglutarate synthases, citrate cleaving enzymes were absent from the SAR324 genomes. Examples of prokaryotes growing autotrophically by means of a rTCA cycle, without known citrate cleaving enzymes, have been previously reported [52, 53]. At Station ALOHA, the balance of usage between the oxygen tolerant CBB cycle and the oxygen sensitive rTCA cycle would be particularly interesting to explore in the limited oxygen zone (LOZ, water mass with oxygen concentration below 60 µmol/kg) that occurs between 750 and 1000 m (Supp Figure 2) which is also the depth of maximal abundance for SAR324.

Genome-encoded nutrient uptake capacities reflect the balance between autotrophy and heterotrophy in SAR324 ecotypes throughout the water column. While few SAR324 genes encoding ABC transporters for sugars were found in aphotic zone (relative to those for metals), SAR324 ecotypes from the euphotic zone possessed genes supporting a greater capacity to transport and incorporate simple and complex forms of saccharides. This depth-specific differentiation in transporters may indicate that the autotrophic lifestyle is more prevalent in the dark ocean for SAR324, and less so in the euphotic zone.

Sulfur-dependent carbon fixation may be ecologically relevant and has been estimated to account for almost half of the total dark carbon fixation occurring in marine sediments [54, 55]. In the water column, all SAR324 appear to be capable of oxidizing sulfur compounds to support their energy metabolism. SAR324, like most known aerobic sulfur-oxidizing bacteria appear capable of considerable metabolic plasticity and encode the machinery for the utilization of diverse sources of mineral or organic carbon sources using different sulfur compounds as electron donors [56].

SAR324 dwelling in the euphotic zone exhibited another alternative energy source, namely sunlight. SAR324 ecotypes from above 200 m encoded two different types of opsins (PR-like and XR-like) that are known to function as light-activated proton pumps enabling cells to generate a proton motive force from light [38]. Despite the lack of a chemoautotrophic signature in SAR324 found above 200 m, they likely engage in photoheterotrophic lifestyles in the euphotic zone. In line with previous reports from surface waters at Station ALOHA [57], rhodopsin genes from SAR324 exhibited a diel pattern in transcriptional activity peaking at dawn [24, 57]. Interestingly, only the gene coding for XR-like type, which is present only in surface ecotype, showed a strong diel pattern in transcriptional activity but not the one coding for the canonical PR type.

SAR324 ecological and metabolic variability appears to reflect the steep biotic, physical, and chemical gradients and perennial stratification of water masses in the water column [7]. At the surface the sunlit, warm and low nutrient environment is dominated by picophotoautotrophs (*Prochlorococcus* spp.), and streamlined heterotrophs year-round [23, 49]. In these highly productive but nutrient limited surface microbiomes, SAR324 exists in relatively low abundance yet persists throughout the year. As with some other bacterial counterparts [58–60], the phototrophic capability may be a key in their persistence.

The SAR324 ecotype dwelling in the sharp biotic and abiotic transition occurring in the twilight zone, displayed specific metabolic features divergent from those inhabiting the upper and the lower layers. Unlike the general trend of the steep increase in microbial genomes GC percent (GTZ) occurring at this depth [23], SAR324 showed an opposite GC trend with ca 4% lower values below the GTZ (Supp Figure 11) and seems to be the only bacterial clade following this trend [23]. As underlined in this study, the ability to access and assimilate nitrogen contained in organic compounds may explain that nitrogen-driven changes in GC are not observed for SAR324 clade.

In the bathypelagic, SAR324 clade is among the more abundant taxa, together with SAR11, SAR406, *Nitrospina* spp., SAR202 and ammonia-oxidizing Archaea [49]. Distant from sunlight-driven productivity, chemolithotrophy may supplement SAR324 energetic requirements, in addition to chemoorganoheterotrophy. Consistent with this hypothesis, chemolithotrophic microbial activities have been detected in the open ocean mesopelagic zone in the NPSG [17, 61–64]. Tenfold more abundant than in surface waters, deep SAR324 ecotype found below 500 m appear to be highly adapted to the conditions encountered in the dark ocean and are more uniform in their genome features. SAR324 emerges as a key species in the dark ocean microbiome, as indicated by its high abundance and chemoautotrophic abilities.

## Conclusions

The combination of pangenomics of locally relevant population genomes, along with metagenomic and metatranscriptomic time-series, revealed ecogenomic patterns in the phylogeny, metabolism, and ecology of the ubiquitous SAR324 clade. Confirming the presence of SAR324 from the surface to the abyss at Station ALOHA, the metapangenomic approach revealed a cryptic diversity within the same clade with a clear depth and time distribution of ecotypes. Phylogenomic subclade delineation, ecotype distribution, genomic features, and metabolic capabilities showed strong congruence in groupings that highlight the metabolic differentiation and ecological partitioning among clusters of genomes within the same phylogenetic clade.

The four SAR324 ecotypes identified in this study displayed striking divergence in genome structure and metabolic capabilities that were specific to the discrete depth zones that they inhabit. This genomic differentiation may in part explain the ecological success of SAR324 clade across drastically changing microbiomes encountered within the ocean’s interior. SAR324 genomic and metabolic plasticity are probably the key to the ubiquity of this clade within the marine ecosystem.

## Methods

### Sample collection

Seawater from three size fractions (no filtration, 0.03–0.2 µm, and > 0.2 µm) were sampled during 2 cruises (December 2015 and May 2016) from 7 depths (25, 125, 200, and 500 m during the December cruise and 750, 1000, and 4000 m during the May cruise) at Station ALOHA (22° 45′ N, 158° W).

During the December 2015 cruise, seawater samples were collected in 50-ml conical tubes from carboys used to collect water from the rosette, for downstream single-cell isolation. For each sample depth, ten 1-ml aliquots of unfiltered seawater were subsampled from the 50 ml conical tube and 250 ml (500 ml for 4000 m samples) of 0.22 µm (MilliPore SteriPak-GP filter, [2–5]) filtered seawater was concentrated to approximately 1 ml on a 0.03-µm polycarbonate filter using a vacuum manifold, then resuspended by vortexing during glycerol fixation. In May 2016, whole seawater (250 ml for surface and 500 ml for 4000 m samples) was concentrated to approximately 1 ml on a 0.2-µm polycarbonate filter using a vacuum manifold following the same methods. All types of fractionated samples were fixed using 100 µL of cryopreservation solution (autoclaved TFF-ultrafiltered surface seawater from Station ALOHA amended with TE buffer and glycerol) and stored at – 80 °C until further processing.

For metagenome sample preparation, seawater was collected in 15-L carboys from the rosette pooled into a 55-gallon barrel (208.3 L) until full. The entire volume of seawater was 11 µm-prefiltered (Whatman® Grade 1 Qualitative Filter) and collected on a 0.2-µm Millipore® Steripak™-GP filter units amended with 15 ml of sucrose lysis buffer (40 mM EDTA, 50 mM Tris pH 8.3, 0.75 mM Sucrose) and stored at – 80 °C until further processing.

### DNA extraction and purification

DNA extractions of cells on 0.22 µm Steripak filters were performed on ice with sucrose lysis buffer amended with lysozyme (2 mg/mL). The filters were incubated for 30 min at 37 °C before adding proteinase K (0.75 mg/mL) and sodium dodecyl sulfate (SDS, 1%) and then incubated again for 2 h at 55 °C. After gentle lysate removal, samples were purified on a Chemagic MSM robotic lab instrument (Perkin Elmer, Walthamm, MA) using a DNA Saliva Kit (Perkin Elmer, CMG-1037–1, Waltham, MA) and eluted in a MSM compatible deep 96-well plate with a final volume of 100 µL. Upon completion of the purification step, corresponding wells to each respective sample were then pooled together. An aliquot of each pool was taken and set aside for subsequent downstream metagenomic library preparation and sequencing.

### Metagenomic library preparation and DNA sequencing

For each depth from which SAGs were produced, deeply sequenced metagenomes were generated. Metagenomic libraries were prepared using Illumina’s automated NeoPrep instrument with TruSeq Nano DNA library preparation kit (Illumina #NP1011001) following guidelines using an input of 25 ng of sheared genomic DNA. Libraries were sequenced on a 150-bp paired-end NextSeq500/550 High Output v2 reagent kit (Illumina #FC4042004).

Metagenomes were prepared and assembled using a custom workflow (https://github.com/jmeppley/workflows/tree/Aloha_2_0.02) executed by Snakemake ([65] v3.11.0). First, suspect reads were removed with two passes of BBDuk (v36.84, https://jgi.doe.gov/data-and-tools/bbtools). The first pass removed sequencing adapters (options: “ktrim = r k = 23 mink = 11 hdist = 1 tbo tpe tbo tpe”), and the second removed phix and extreme GC values (options: “k = 27 hdist = 1 qtrim = rl trimq = 17 cardinality = t 'mingc = 0.05 maxgc = 0.95”). Next, sequencing errors were corrected and low abundance kmers removed using BFC ([66], vr181, options: “-k 21 -1”). In the last cleaning step, low-quality sequence was removed from read ends and short reads were dropped using Trimmomatic ([67], v35, options: “LEADING:10 TRAILING:10 MINLEN:50”). Finally, the resulting 696 M cleaned reads from 7 metagenomes were assembled in separate assemblies totaling 11.4 M contigs using MetaSPAdes ([68], v3.10.1, options: “-k 21,33,55,77,99,127”).

### Single-cell amplified genome sequencing

Single-cell amplified genomes (SAGs) were generated at the DOE Joint Genome Institute (JGI) following the methods outlined previously by Rinke et al. [69]. Briefly, individual cells sorted on a BD Influx™ (BD Biosciences) were treated with Ready-Lyse™ Lysozyme (Epicentre; 5 U/μl) for 15 min at room temperature before the addition of lysis solution. For the whole water samples from 25 and 125 m, chlorophyll-containing cells were selectively sorted away. Whole-genome amplification was performed with the REPLI-g® Single Cell Kit (Qiagen) in 2-μl reactions set up with an Echo acoustic liquid handler (Labcyte™). Amplification reactions were terminated after 6 h. PCR amplification and Sanger sequencing of a ~ 470-bp region of the 16S rRNA gene (using primers 926wF (5′-AAACTYAAAKGAATTGRCGG-3′) and 1392R (5′-ACGGGCGGTGTGTRC-3′)[69] was used to assign a preliminary taxonomic identification to each of the SAGs. For each isolated genome selected, a 2 × 151 bp NextSeq Illumina® library was constructed and sequenced.

Reads were quality-filtered, trimmed, and assembled. Briefly, BBTools suite (v36.84, https://jgi.doe.gov/data-and-tools/bbtools), especially BBDuk, BBMap, BBMerge commands, was used to remove contaminants, trim reads that contained adapter sequences, and remove reads containing undefined bases (“N”) or having a minimum length of 51 bp. Reads that mapped to masked human, cat, dog, and mouse references at 93% identity or that mapped to masked common microbial contaminants were filtered out. Remaining reads were assembled in draft genomes using SPAdes ([70], v3.9.0, option: “–phred-offset 33 -t 16 -m 120 –sc –careful -k 25,55,95 –12”), and contigs with low-coverage or too short (< 2 kb) were excluded using BBTools and a decontamination was performed using ProDeGe ([71], v2.2). The quality of the resulting draft genomes was assessed automatically and MISAG quality standards were applied [72]. All metadata associated with each SAGs are available on JGI-IMG portal accessible using permalinks listed in Supp Table 2.

### Creation of locally relevant SAR324 population genomes

Using the IMG/M system [73], SAGs belonging to the SAR324 clade were identified. To analyze functions and to cope with the low completeness and the high fragmentation of obtained SAGs (Supp Table 1), population genomes were constructed. First, average nucleotide identity (ANI) for all pairs of SAGs was computed using pyANI [74]. Groups of draft genomes were clustered by single linkage at 95% of homology (SLc) using *hclust* function from the R package *stats* ([75], v3.4.2). Genes, and putative proteins produced, were predicted from contigs of each genome using Prodigal ([76], v2.6.3). A catalog of dereplicated genes (95% of nucleic acid identity, alignment covering at least 90% of the gene length) for each ANI-cluster was computed using CD-HIT-EST ([77], v4.7, options: “-c 0.95 -aS 0.9 -g 1 -r 1 -d 0 -G 0 -M 0 -T 24”).

In parallel, genomes belonging to SAR324 clade were retrieved from public databases. Ten universal single-copy genes were extracted from them and SAGs using fetchMGs [78] and homologs in NCBI prokaryote genomes were searched using blastN [79] and filtered using gene-specific bitscore according NCBI CDD structure (*COG0012*, 336.459; *COG0016*, 271.407; *COG0018*, 494.85; *COG0172*, 267.927; *COG0215*, 427.8; *COG0495*, 358.516; *COG0525*, 655.085; *COG0533*, 311.045; *COG0541*, 525.175; *COG0552*, 306.875). Quality of all retrieved genomes was assessed using CheckM ([80], v1.0.7). Candidate reference genomes identified using this approach were added to the collection and an ANI matrix was computed using pyANI. Groups of reference genomes were clustered by complete linkage at 95% ANI and population genomes were generated as previously described using complete linkage (CLc). To reduce complexity of dataset to locally pertinent genomes only, metagenomic reads from the seven depths of isolation were mapped on both types of population genomes (SLc and CLc) using bowtie2 ([81], v2.3.4.1), SAM files were processed to binary alignment map (BAM) using samtools ([82], v1.8). Reference population genomes not relevant at Station ALOHA (i.e., with no reads mapped) have been discarded from downstream analysis. The final dataset was composed of 18 population genomes pooling together with the 14 SAGs generated in this study, 28 MAGs and 6 SAGs from public databases (Supp Table 1).

### Metapangenome computation

Locally relevant SAR324 population genomes were imported under Anvi’o ([83], v5.1) and processed following recommendations for pangenomic analysis [43]. Briefly, each genome was imported into an Anvi’o database (*anvi-gen-contigs-database*) for which were added the HMM profiles (*anvi-run-hmms*), COGs annotations (*anvi-run-ncbi-cogs –sensitive*) of each gene, and BAM profiles (*anvi-init-bam* and *anvi-profile*). All Anvi’o databases were merged into a genomes collection (*anvi-merge* and *anvi-import-collection*) and *anvi-pan-genome* command was used to generate pangenome. Genes identity was assessed using blastP with weak matches between amino acid sequences removed using the minbit heuristic (*–minbit 0.5*). Genes were clustered into orthogroups using MCL algorithm [84] with an inflation of 10. Euclidean distance and ward linkage to organize gene clusters and genomes were used by default. ANI between all selected population genomes was also computed using *anvi-compute-ani*. They were classified using average linkage and ordered in a newick tree using *hclust* and *as.phylo* functions from the R packages *stats* and *ape*, respectively. Orthogroups of proteins were extracted (*anvi-summarize*) and their mean abundance in metagenomes was calculated using *pandas* Python library ([85], v0.23.1) and imported under Anvi’o using *anvi-import-misc-data* function.

### Functional analyses

Orthogroups of proteins were annotated using multiple reference databases (COG [86], KEGG [87] v84.0 and Pfam [88] v30.0) using annotation.gene_catalog.snake script (available at https://github.com/jmeppley/workflows/blob/master/annotation.gene_catalog.snake). For each protein orthogroups, abundance and transcriptional activity in depth and time were retrieved from quality-controlled metagenomic and metatranscriptomic reads mapping on closest relatives (80% of identity and 70% of coverage) in genes catalogs from Aylward et al., Mende et al., and Boeuf et al. [23, 24, 36]. Metabolic pathways were reconstructed using KEGG mapper (https://www.genome.jp/kegg/mapper.html, v3.1) using genes with assigned KO identifier. Heatmap and classification of metabolic pathways distribution (scaled from 0 to 1 for each pathway) were assessed using *pandas*, *seaborn* ([89], v0.8), and *matplotlib* ([90], v2.2.3) Python libraries. Contigs comprising genes of interest were retrieved from partial genomes, gene predicted and annotated using Prodigal and Prokka ([91], v1.12), respectively. Synteny maps were generated using easyfig [92] with a similarity between CDS assessed using blastN.

## Supplementary Information

**Additional file 1: Supplementary Table 1.** Features of genomes used in this study.

**Additional file 2: Supplementary Table 2.** Links to repository of SAGs isolated from this study.

**Additional file 3: Supplementary Table 3.** Orthogroups distribution among ecotypes.

**Additional file 4: Supplementary Figure 1.** Venn diagrams of SAR324 genes pooled either by subclade (A) or by ecotype (B). The total of genes shared among the genomes constituting the subclade or the ecotype, are displayed in italic grey below the number of genes unique to the subclade or the ecotype. Venn diagrams have been generated from http://bioinformatics.psb.ugent.be/webtools/Venn.

**Additional file 5: Supplementary Figure 2.** Depth distribution of SAR324 average coverage in the same samples from which the SAGs have been isolated (black) and in HOT time-series (grey). Depth distribution of physical and chemical parameters at each month of 2016 are displayed in red and the average profile in blue. T: temperature, S: salinity, O: oxygen concentration, NO_2_+NO_3_: Nitrite and nitrate concentration, P: phosphorus concentration.

**Additional file 6: Supplementary Figure 3.** Depth distribution of SAR324 ABC-transporter at Station ALOHA.

**Additional file 7: Supplementary Figure 4.** Placement of SAR324 16S rRNA coding genes (red) into the SILVA 132 reference tree. Subclades as defined by the ANI in this study are displayed by the inner brackets. Outside brackets denote the official classification as based on the SILVA database. 16S rDNA sequences retrieved from population genomes were aligned using SINA and placed into the reference tree using ARB_add_by_parcimony as implemented in ARB software. Genes are identified as follow: Population genome identifier (this study) | GenBank assemblies (GCA) identifier | genome description.

**Additional file 8: Supplementary Figure 5.** Comparison between phylogenetic tree of 16S rRNA coding genes (left) and ANI classification (right). Phylogenetic tree of 16S rDNA genes was inferred from a MUSCLE alignment using Maximum Likelihood and General Time Reversible model with MEGA X software.

**Additional file 9: Supplementary Figure 6.** Placement of SAR324 rhodopsin genes (red) into the MicRhoDE reference tree. Rhodopsin protein sequences retrieved from SAR324 population genomes were aligned on MicRhoDE reference alignment using MAFFT --addfragments and backtranslated using pal2nal software before being placed into the reference tree using ARB_add_by_parcimony as implemented in ARB software. Genes are identified as follow: Population genome identifier | orthogroups cluster identifier | gene identifier.

**Additional file 10: Supplementary Figure 7.** Protein alignment of rhodopsins identified in SAR324 population genomes and closest relatives retrieved from HOT time-series metagenomes. Amino acid residue motifs involved in ion pumping, opsin fixation and spectral tuning are highlighted by black rectangles. Proteorhodpsin-like sequences are displayed in blue and Xanthorhodopsin-like sequences in green. Amino acid residues are colored according to properties and conservation of residues (ClustalX). Consensus sequence and colored bars of sequence conservation have been created using SnapGene Viewer v.4.2.6. Predicted secondary and tertiary structures of rhodopsins have been predicted using RaptorX server.

**Additional file 11: Supplementary Figure 8.** Synteny map of the genic neighborhood of Xanthorhodopsin-like coding genes retrieved in SAR324 genomes. Target gene is displayed in red, enzyme-coding gene in orange, hypothetical enzyme-coding gene in yellow, transporter-coding gene in green, protein-coding gene in grey and hypothetical coding gene in white. tRNA are displayed by black bars. Contigs are identified as follow: Population genome identifier - GenBank assemblies (GCA) identifier (contig identifier). Name background was colored according to subclades.

**Additional file 12: Supplementary Figure 9.** Synteny map of the genic neighborhood of Proteorhodopsin-like coding genes retrieved in SAR324 genomes. Target gene is displayed in red, enzyme-coding gene in orange, hypothetical enzyme-coding gene in yellow, transporter-coding gene in green, protein-coding gene in grey and hypothetical coding gene in white. tRNA are displayed by black bars. Contigs are identified as follow: Population genome identifier - GenBank assemblies (GCA) identifier (contig identifier). Name background was colored according to subclades.

**Additional file 13: Supplementary Figure 10.** Phylogenetic reconstruction of SAR324 RuBisCO types. RuBisCO protein sequences retrieved from SAR324 population genomes (in red) were aligned on type references from Tabita et al 2007 using MUSCLE. Neighbor Joining phylogenetic reconstruction using a Poisson model; Bootstrapping of 1000.

**Additional file 14: Supplementary Figure 11.** Box plot of GC percentage of SAR324 ecotypes.

## Data Availability

The datasets supporting the conclusions of this article are available in the JGI Integrated Microbial Genomes & Microbiomes (IMG/M) database repository, https://img.jgi.doe.gov/cgi-bin/m/main.cgi. List of permalinks pointing to JGI-IMG webpages for each SAG generated for this study is included within additional tables of this article (Supp. Table 2). In addition to provide assembled sequences, all metadata (samples location and depth of isolation, sequencing procedure and technology, bioinformatic processing and quality control, and genomic statistics) associated with SAGs are available there. Additional genomes used to build pangenome have been retrieved from NCBI, ftp://ftp.ncbi.nlm.nih.gov/genomes/genbank/bacteria/. Metagenome and metatranscriptome data are available in the NCBI SRA projects PRJNA352737 and PRJNA358725. All other data products associated with this study are accessible online: population genomes, https://doi.org/10.6084/m9.figshare.14573751.v1, and Anvi’o databases, https://doi.org/10.6084/m9.figshare.14573046.v1.

## Abbreviations

ALOHA: A Long-term Oligotrophic Habitat Assessment
ANI: Average nucleotide identity
APS: Adenosine 5′-phosphosulfate
BAM: Binary Alignment Map
CBB: Calvin-Benson-Basham
CLc/SLc: Complete linkage cluster/single linkage cluster
COG: Clusters of orthologous groups
DCM: Deep chlorophyll maximum
DIC: Dissolved inorganic carbon
DMSP/DMSO: Dimethylsulfoniopropionate/dimethyl sulfoxide
DOE-JGI: US Department Of Energy Joint Genome Institute
EDTA: Ethylenediaminetetraacetic acid
GTZ: Genomic transition zone
HMM: Hidden Markov model
ITS: Internal transcribed spacers
IMG/M: JGI Integrated Microbial Genomes & Microbiomes
KEGG: Kyoto Encyclopedia of Genes and Genomes
KO: KEGG Orthology
LOZ: Limited oxygen zone
MAG: Metagenomic assembled genome
NPSG: North Pacific Subtropical Gyre
OMZ: Oxygen minimum zone
PCR: Polymerase chain reaction
PEP: Phosphoenolpyruvate
RuBisCO: Ribulose-bisphosphate carboxylase
RLP: RuBisCO-like protein
SAG: Single-cell amplified genome
SDS: Sodium dodecyl sulfate
SOX/TOMES: Sulfur oxidation/thiosulfate oxidation multienzymes system
SSU: Small subunit
TCA: Tricarboxylic acid cycle
THF: Tetrahydrofolic acid cycle
