# Supplementary figures and images for: Metapangenomics reveals depth-dependent shifts in metabolic potential for the ubiquitous marine bacterial SAR324 lineage
Source: Microbiome. 2021 Aug 13;9:172. doi: 10.1186/s40168-021-01119-5 (PMC8364033; doi:10.1186/s40168-021-01119-5)

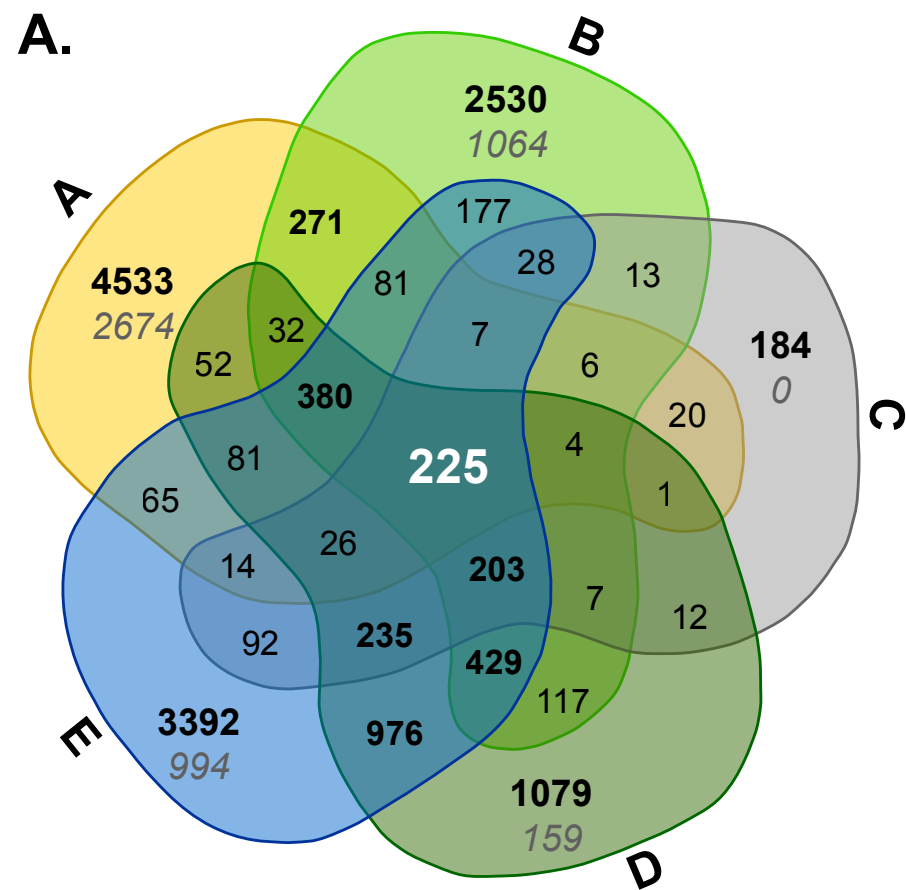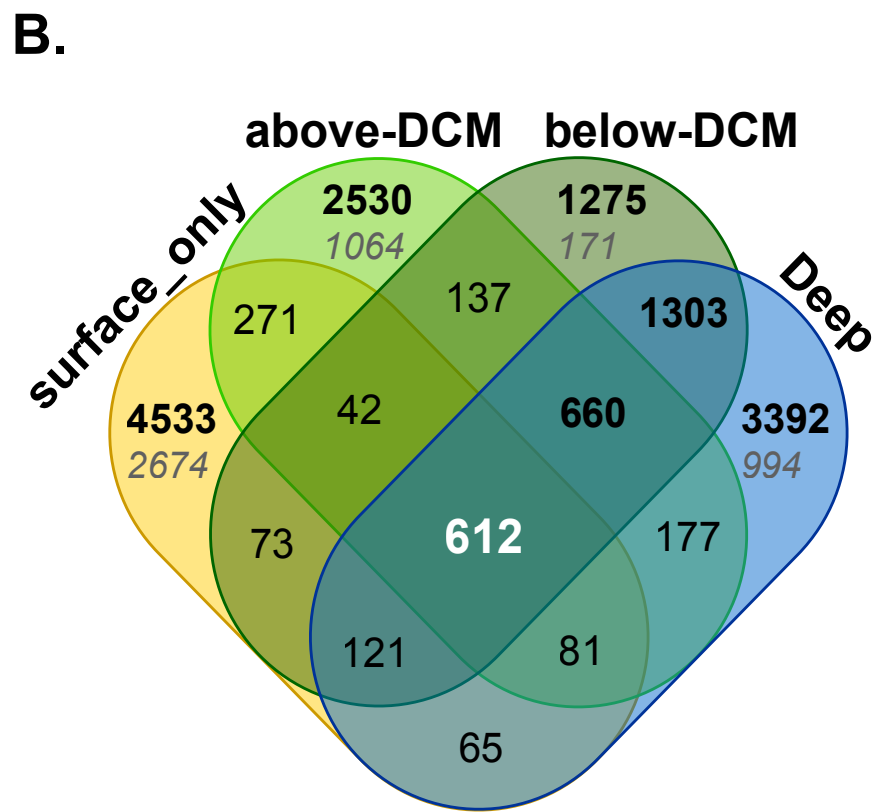

Supplement: Supplementary file 5 — Additional file 4: Supplementary Figure 1. Venn diagrams of SAR324 genes pooled either by subclade (A) or by ecotype (B). The total of genes shared among the genomes constituting the subclade or the ecotype, are displayed in italic grey below the number of genes unique to the subclade or the ecotype. Venn diagrams have been generated from http://bioinformatics.psb.ugent.be/webtools/Venn. [file 40168_2021_1119_MOESM5_ESM.pdf]

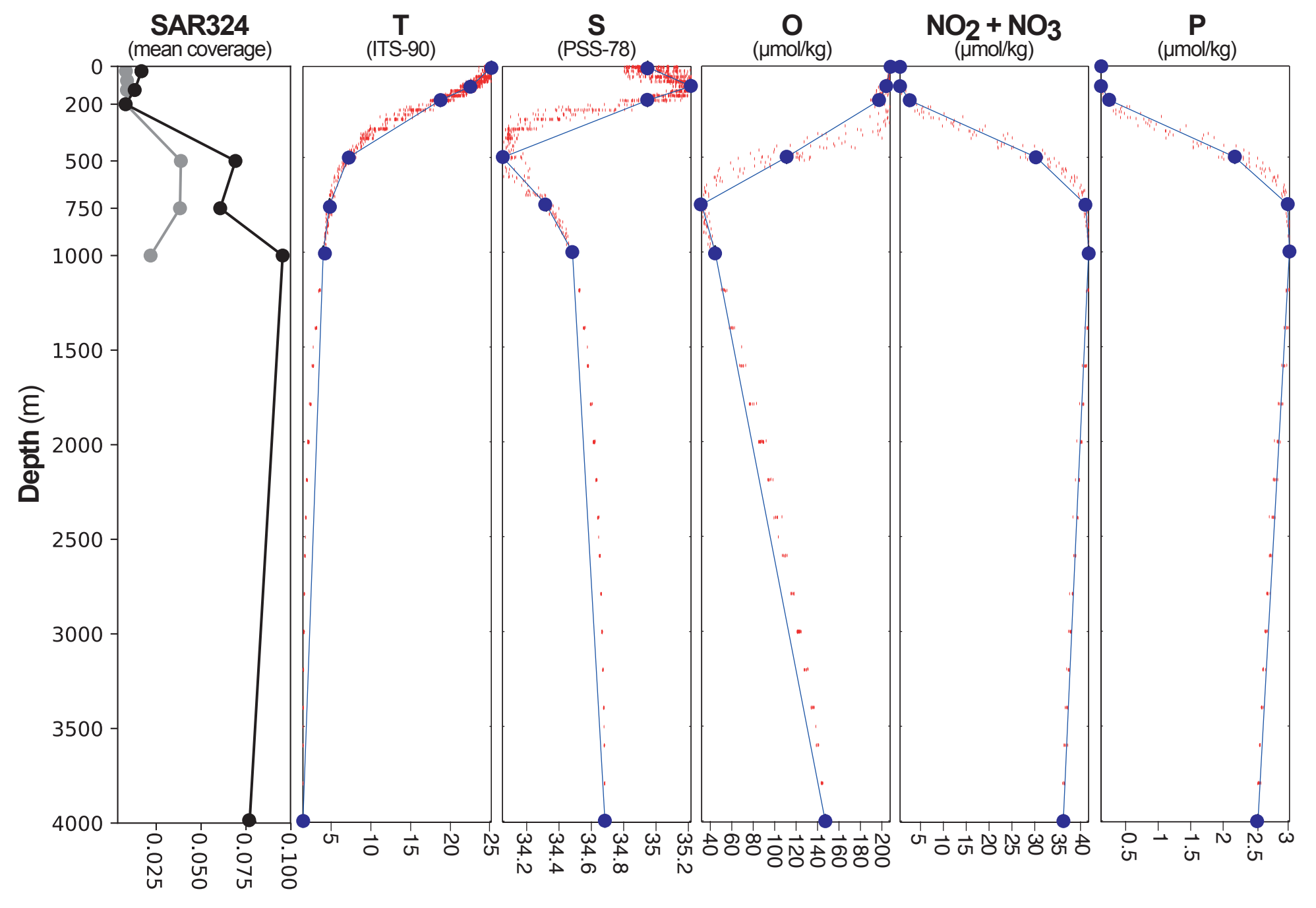

Supplement: Supplementary file 6 — Additional file 5: Supplementary Figure 2. Depth distribution of SAR324 average coverage in the same samples from which the SAGs have been isolated (black) and in HOT time-series (grey). Depth distribution of physical and chemical parameters at each month of 2016 are displayed in red and the average profile in blue. T: temperature, S: salinity, O: oxygen concentration, NO2+NO3: Nitrite and nitrate concentration, P: phosphorus concentration. [file 40168_2021_1119_MOESM6_ESM.pdf]

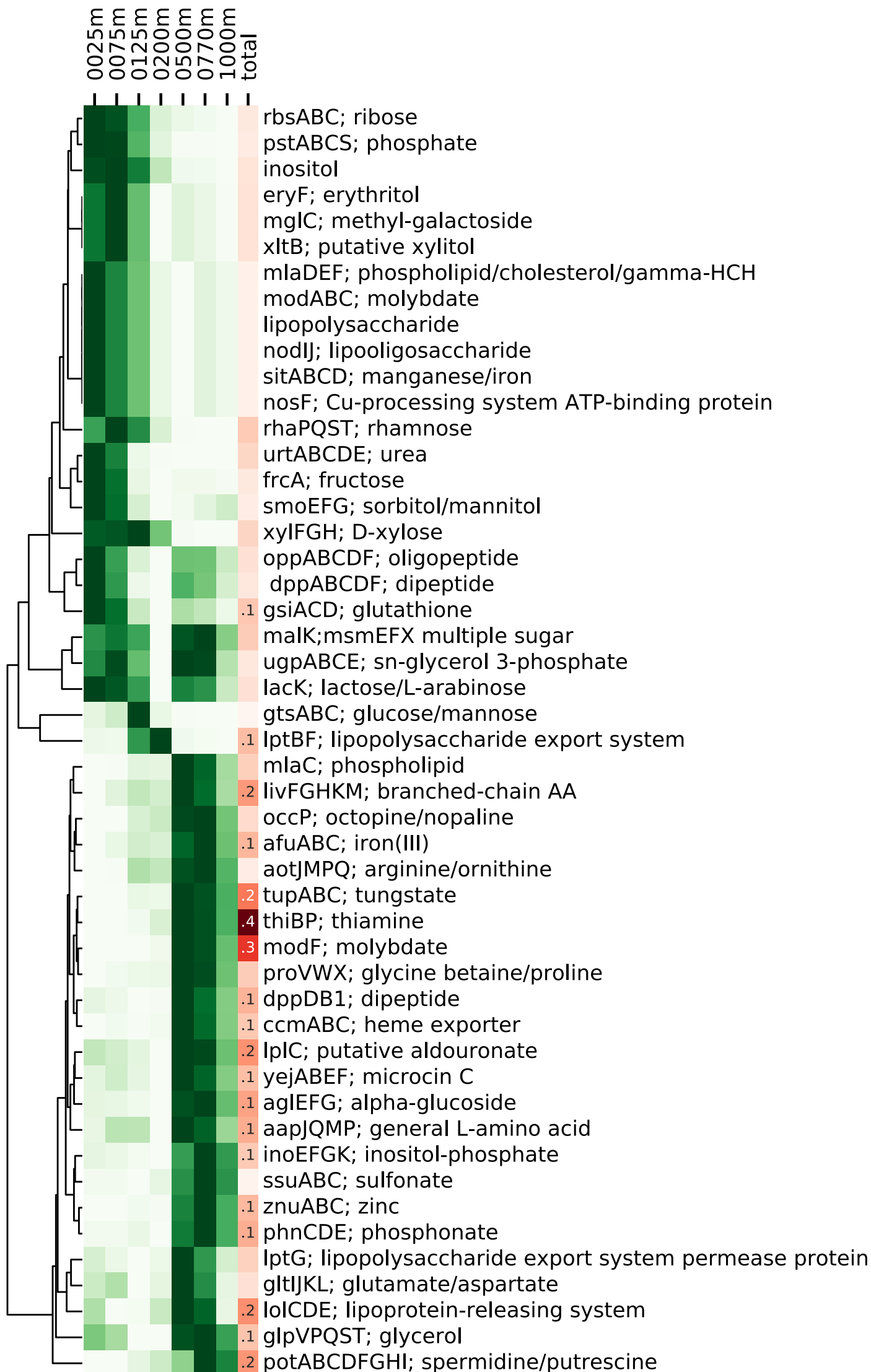

Supplement: Supplementary file 7 — Additional file 6: Supplementary Figure 3. Depth distribution of SAR324 ABC-transporter at Station ALOHA. [file 40168_2021_1119_MOESM7_ESM.pdf]

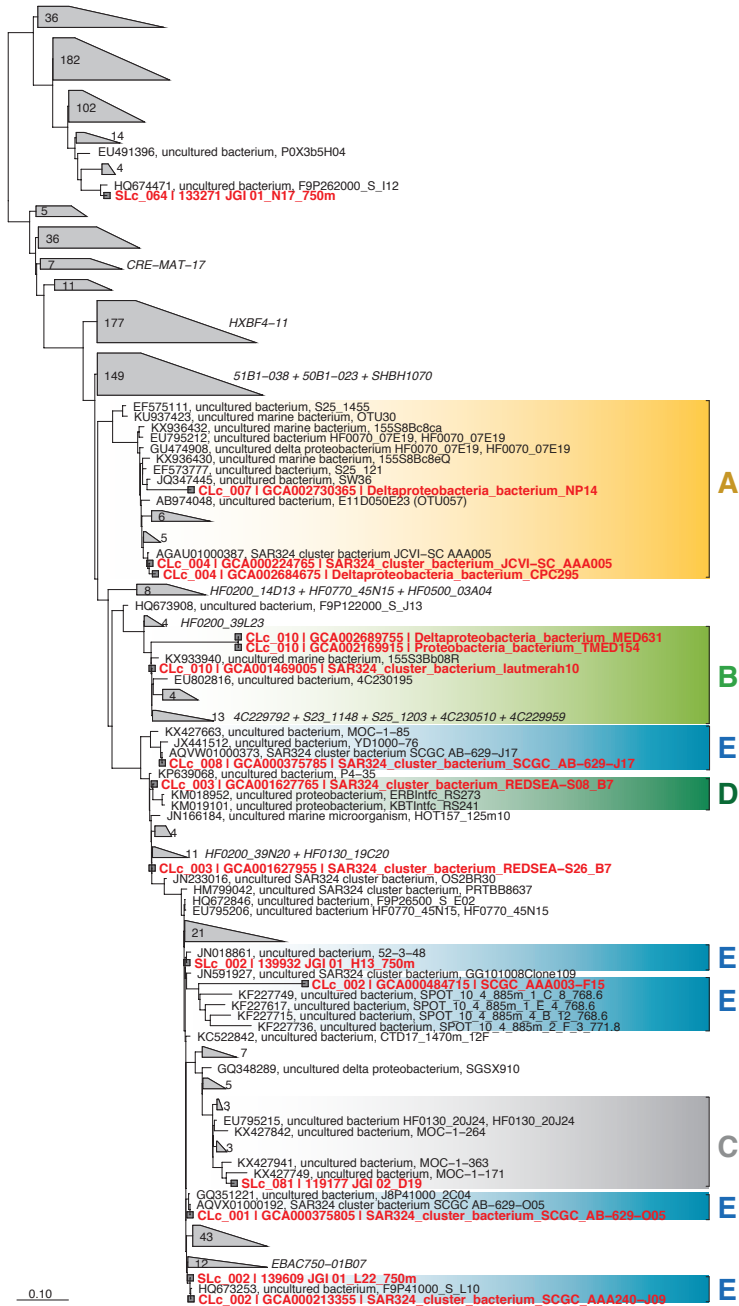

Supplement: Supplementary file 8 — Additional file 7: Supplementary Figure 4. Placement of SAR324 16S rRNA coding genes (red) into the SILVA 132 reference tree. Subclades as defined by the ANI in this study are displayed by the inner brackets. Outside brackets denote the official classification as based on the SILVA database. 16S rDNA sequences retrieved from population genomes were aligned using SINA and placed into the reference tree using ARB_add_by_parcimony as implemented in ARB software. Genes are identified as follow: Population genome identifier (this study) | GenBank assemblies (GCA) identifier | genome description. [file 40168_2021_1119_MOESM8_ESM.pdf]

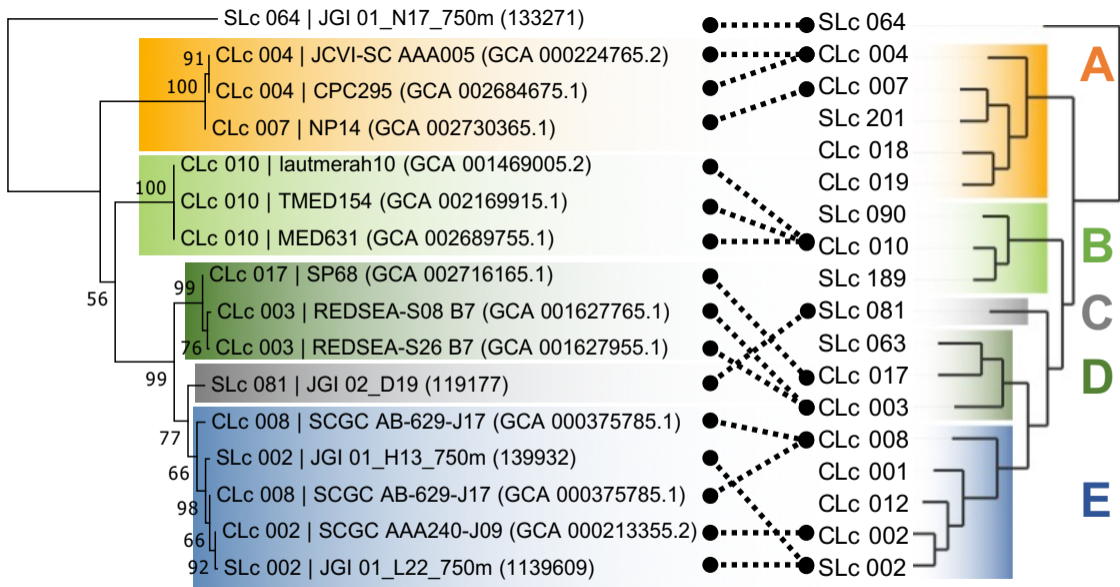

Supplement: Supplementary file 9 — Additional file 8: Supplementary Figure 5. Comparison between phylogenetic tree of 16S rRNA coding genes (left) and ANI classification (right). Phylogenetic tree of 16S rDNA genes was inferred from a MUSCLE alignment using Maximum Likelihood and General Time Reversible model with MEGA X software. [file 40168_2021_1119_MOESM9_ESM.pdf]

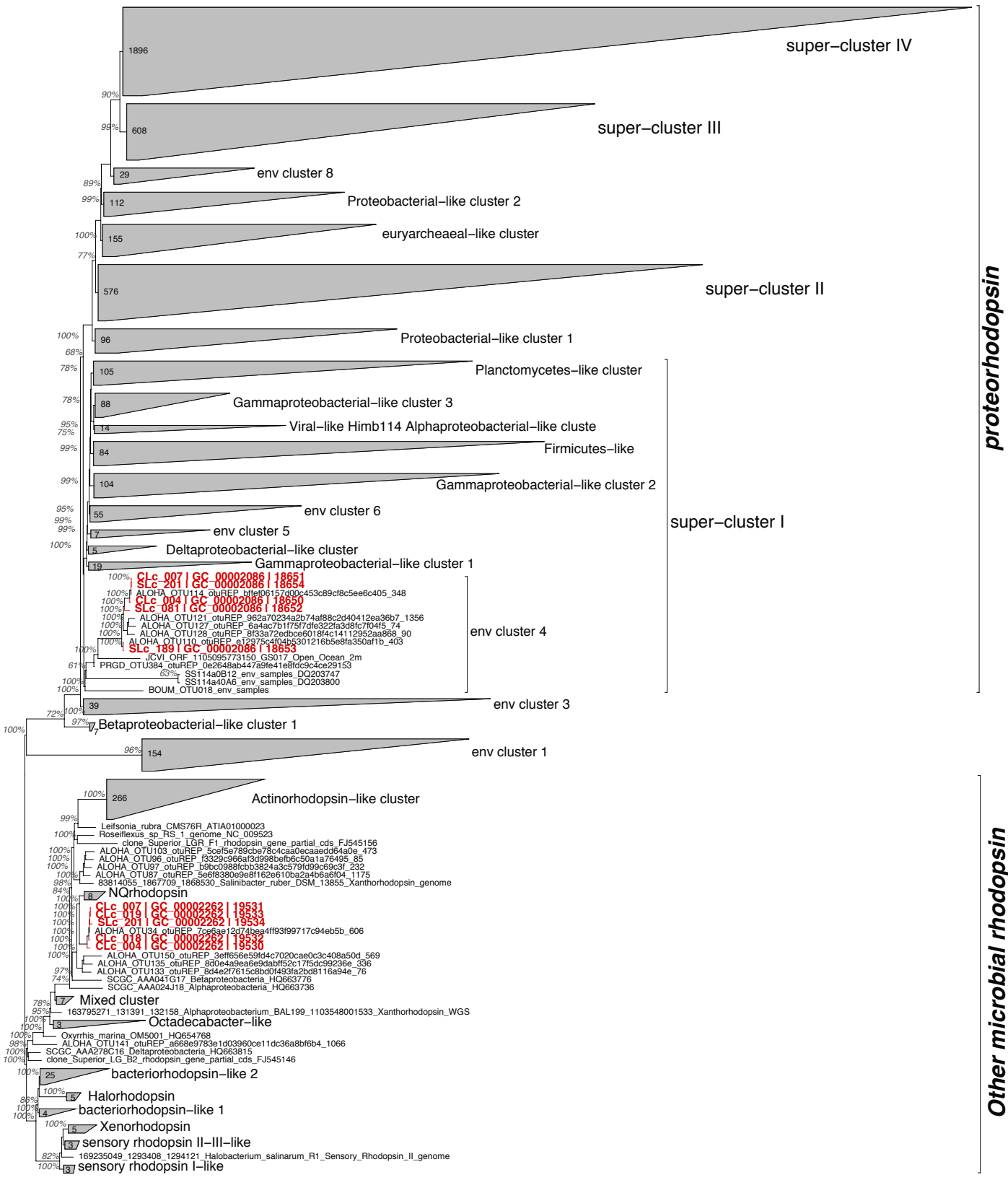

proteorhodopsin

Other microbial rhodopsin

Supplement: Supplementary file 10 — Additional file 9: Supplementary Figure 6. Placement of SAR324 rhodopsin genes (red) into the MicRhoDE reference tree. Rhodopsin protein sequences retrieved from SAR324 population genomes were aligned on MicRhoDE reference alignment using MAFFT --addfragments and backtranslated using pal2nal software before being placed into the reference tree using ARB_add_by_parcimony as implemented in ARB software. Genes are identified as follow: Population genome identifier | orthogroups cluster identifier | gene identifier. [file 40168_2021_1119_MOESM10_ESM.pdf]

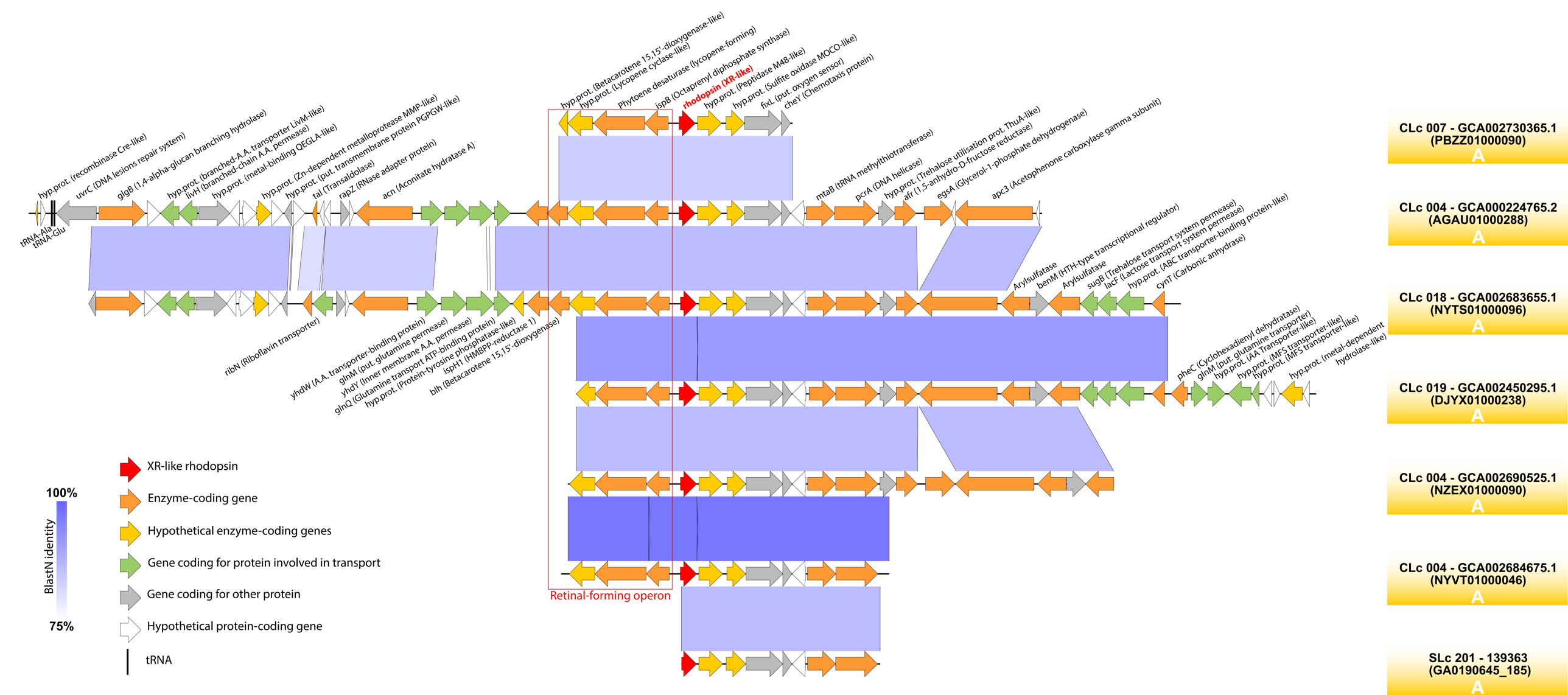

Supplement: Supplementary file 12 — Additional file 11: Supplementary Figure 8. Synteny map of the genic neighborhood of Xanthorhodopsin-like coding genes retrieved in SAR324 genomes. Target gene is displayed in red, enzyme-coding gene in orange, hypothetical enzyme-coding gene in yellow, transporter-coding gene in green, protein-coding gene in grey and hypothetical coding gene in white. tRNA are displayed by black bars. Contigs are identified as follow: Population genome identifier - GenBank assemblies (GCA) identifier (contig identifier). Name background was colored according to subclades. [file 40168_2021_1119_MOESM12_ESM.pdf]

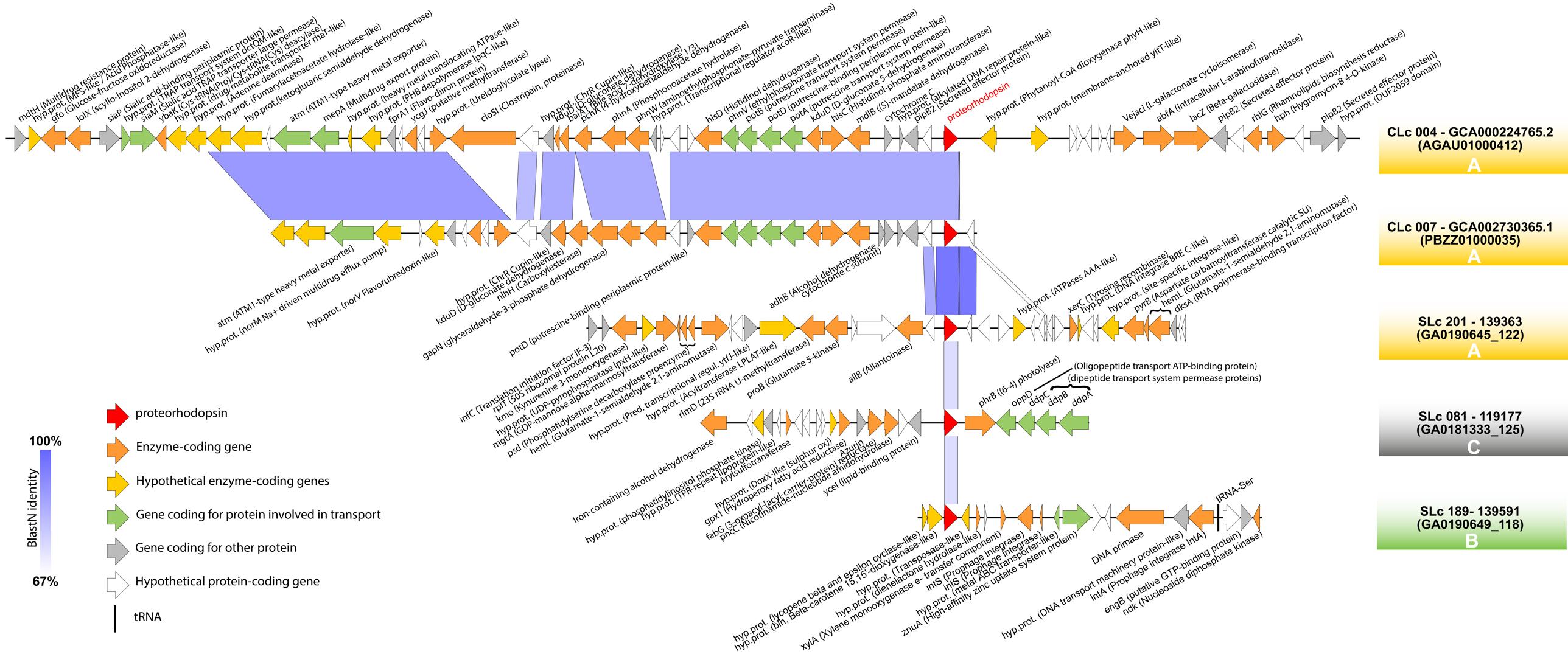

Supplement: Supplementary file 13 — Additional file 12: Supplementary Figure 9. Synteny map of the genic neighborhood of Proteorhodopsin-like coding genes retrieved in SAR324 genomes. Target gene is displayed in red, enzyme-coding gene in orange, hypothetical enzyme-coding gene in yellow, transporter-coding gene in green, protein-coding gene in grey and hypothetical coding gene in white. tRNA are displayed by black bars. Contigs are identified as follow: Population genome identifier - GenBank assemblies (GCA) identifier (contig identifier). Name background was colored according to subclades. [file 40168_2021_1119_MOESM13_ESM.pdf]

GC %

Surface only

Above DCM

Below DCM

Deep

48.0  
46.0  
44.0  
42.0  
40.0  
38.0  
36.0  
34.0  
32.0

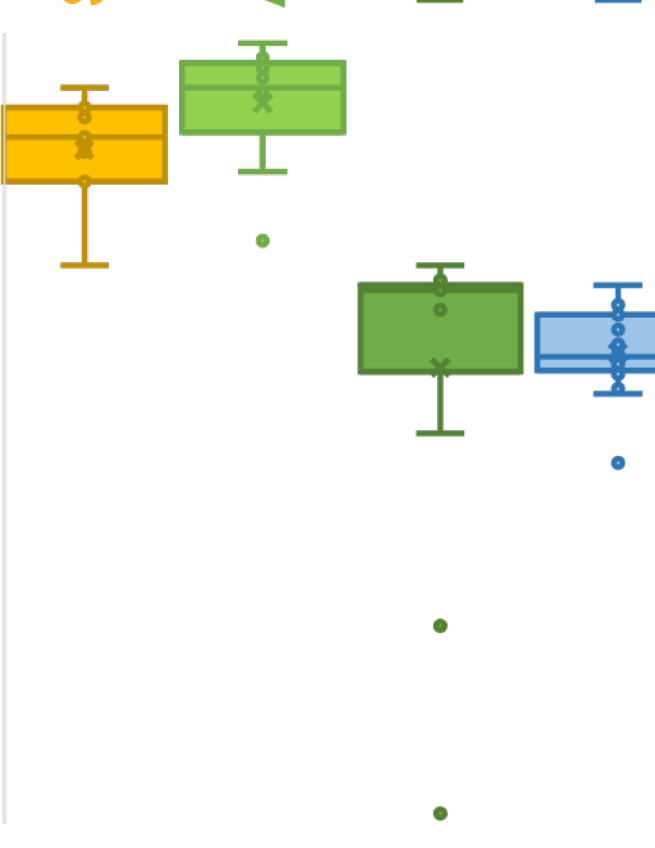

Supplement: Supplementary file 15 — Additional file 14: Supplementary Figure 11. Box plot of GC percentage of SAR324 ecotypes. [file 40168_2021_1119_MOESM15_ESM.pdf]
