# Supplementary material for: Metapangenomics reveals depth-dependent shifts in metabolic potential for the ubiquitous marine bacterial SAR324 lineage
Source: Microbiome. 2021 Aug 13;9:172. doi: 10.1186/s40168-021-01119-5 (PMC8364033; doi:10.1186/s40168-021-01119-5)
Supplement: Supplementary file 11 — Additional file 10: Supplementary Figure 7. Protein alignment of rhodopsins identified in SAR324 population genomes and closest relatives retrieved from HOT time-series metagenomes. Amino acid residue motifs involved in ion pumping, opsin fixation and spectral tuning are highlighted by black rectangles. Proteorhodpsin-like sequences are displayed in blue and Xanthorhodopsin-like sequences in green. Amino acid residues are colored according to properties and conservation of residues (ClustalX). Consensus sequence and colored bars of sequence conservation have been created using SnapGene Viewer v.4.2.6. Predicted secondary and tertiary structures of rhodopsins have been predicted using RaptorX server. [file 40168_2021_1119_MOESM11_ESM.pdf]

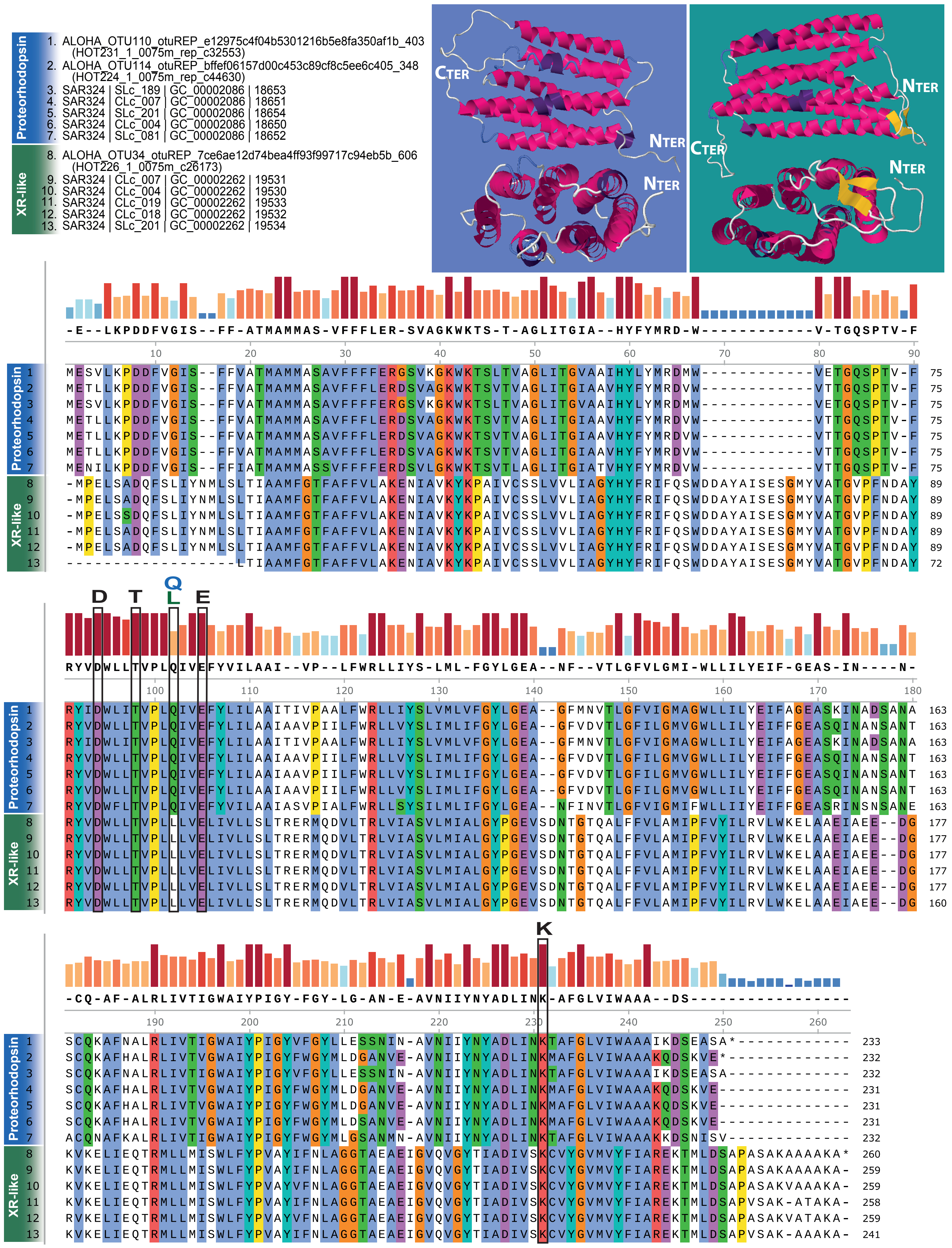

Proteorhodopsin
1. ALOHA_OTU110_otuREP_e12975c4f04b5301216b5e8fa350af1b_403 (HOT231_1_0075m_rep_c32553)
2. ALOHA_OTU114_otuREP_bffef06157d00c453c89cf8c5ee6c405_348 (HOT224_1_0075m_rep_c44630)
3. SAR324 | SLc_189 | GC_00002086 | 18653
4. SAR324 | CLc_007 | GC_00002086 | 18651
5. SAR324 | SLc_201 | GC_00002086 | 18654
6. SAR324 | CLc_004 | GC_00002086 | 18650
7. SAR324 | SLc_081 | GC_00002086 | 18652
XR-like
8. ALOHA_OTU34_otuREP_7ce6ae12d74bea4ff93f99717c94eb5b_606 (HOT226_1_0075m_c26173)
9. SAR324 | CLc_007 | GC_00002262 | 19531
10. SAR324 | CLc_004 | GC_00002262 | 19530
11. SAR324 | CLc_019 | GC_00002262 | 19533
12. SAR324 | CLc_018 | GC_00002262 | 19532
13. SAR324 | SLc_201 | GC_00002262 | 19534
CTER
NTER
CTER
NTER
NTER
NTER
-E--LKPDDFVGIS--FF-ATMAMMAS-VFFFLER-SVAGKWKTS-T-AGLITGIA--HYFYMRD-W-----------V-TGQSPTV-F
10 20 30 40 50 60 70 80 90
1 MESVLKPDDFVGIS--FFVATMAMMASAVFFFFERGSVKGKWKTSLTVAGLITGVAAIHYLYMRDMW------------VETGQSPTV-F 75
2 METLLKPDDFVGIS--FFVATMAMMASAVFFFLERDSVAGKWKTSVTVAGLITGIAAVHYFYMRDVW------------VTTGQSPTV-F 75
3 MESVLKPDDFVGIS--FFVATMAMMASAVFFFFERGSVKGKWKTSLTVAGLITGVAAIHYLYMRDMW------------VETGQSPTV-F 75
4 METLLKPDDFVGIS--FFVATMAMMASAVFFFLERDSVAGKWKTSVTVAGLITGIAAVHYFYMRDVW------------VTTGQSPTV-F 75
5 METLLKPDDFVGIS--FFVATMAMMASAVFFFLERDSVAGKWKTSVTVAGLITGIAAVHYFYMRDVW------------VTTGQSPTV-F 75
6 METLLKPDDFVGIS--FFVATMAMMASAVFFFLERDSVAGKWKTSVTVAGLITGIAAVHYFYMRDVW------------VTTGQSPTV-F 75
7 MENILKPDDFVGIS--FFIATMAMMASSVFFFFERDSVLGKWKTSVTLAGLITGIATVHYFYMRDVW------------VTTGQSPTV-F 75
8 -MPELSADQFSLIYNMLSLTIAAMFGTFAFFVLAKENIAVKYKPAIVCSSLVVLIAGYHYFRIFQSWDDAYAISESGMYVATGVPFNDAY 89
9 -MPELSADQFSLIYNMLSLTIAAMFGTFAFFVLAKENIAVKYKPAIVCSSLVVLIAGYHYFRIFQSWDDAYAISESGMYVATGVPFNDAY 89
10 -MPELSSDQFSLIYNMLSLTIAAMFGTFAFFVLAKENIAVKYKPAIVCSSLVVLIAGYHYFRIFQSWDDAYAISESGMYVATGVPFNDAY 89
11 -MPELSADQFSLIYNMLSLTIAAMFGTFAFFVLAKENIAVKYKPAIVCSSLVVLIAGYHYFRIFQSWDDAYAISESGMYVATGVPFNDAY 89
12 -MPELSADQFSLIYNMLSLTIAAMFGTFAFFVLAKENIAVKYKPAIVCSSLVVLIAGYHYFRIFQSWDDAYAISESGMYVATGVPFNDAY 89
13 -----------------LTIAAMFGTFAFFVLAKENIAVKYKPAIVCSSLVVLIAGYHYFRIFQSWDDAYAISESGMYVATGVPFNDAY 72
D T Q L E
RYVDWLLTVPLQIVEFYVILAAI--VP--LFWRLLIYS-LML-FGYLGEA--NF--VTLGFVLGMI-WLLILYEIF-GEAS-IN----N-
100 110 120 130 140 150 160 170 180
1 RYIDWLITVPLQIVEFYLILAAITIVPAALFWRLLIYSLVMLVFGYLGEA--GFMNVTLGFVIGMAGWLLILYEIFAGEASKINADSANA 163
2 RYVDWLLTVPLQIVEFYLILAAIAAVPIILFWRLLVYSLIMLIFGYLGEA--GFVDVTLGFILGMVGWLLILYEIFFGEASQINANSANT 163
3 RYIDWLITVPLQIVEFYLILAAITIVPAALFWRLLIYSLVMLVFGYLGEA--GFMNVTLGFVIGMAGWLLILYEIFAGEASKINADSANA 163
4 RYVDWLLTVPLQIVEFYLILAAIAAVPIILFWRLLVYSLIMLIFGYLGEA--GFVDVTLGFILGMVGWLLILYEIFFGEASQINANSANT 163
5 RYVDWLLTVPLQIVEFYLILAAIAAVPIILFWRLLVYSLIMLIFGYLGEA--GFVDVTLGFILGMVGWLLILYEIFFGEASQINANSANT 163
6 RYVDWLLTVPLQIVEFYLILAAIAAVPIILFWRLLVYSLIMLIFGYLGEA--GFVDVTLGFILGMVGWLLILYEIFFGEASQINANSANT 163
7 RYVDWFLTVPLQIVEFYVILAAIASVPIALFWRLLSYSILMLIFGYLGEA--NFINVTLGFVIGMIFWLLIIYEIFFGEASRINSNSANE 163
8 RYVDWLLTVPLLLVELIVLLSLTRERMQDVLTRLVIASVLMIALGYPGEVSDNTGTQALFFVLAMIPFVYILRVLWKELAAEIAEE--DG 177
9 RYVDWLLTVPLLLVELIVLLSLTRERMQDVLTRLVIASVLMIALGYPGEVSDNTGTQALFFVLAMIPFVYILRVLWKELAAEIAEE--DG 177
10 RYVDWLLTVPLLLVELIVLLSLTRERMQDVLTRLVIASVLMIALGYPGEVSDNTGTQALFFVLAMIPFVYILRVLWKELAAEIAEE--DG 177
11 RYVDWLLTVPLLLVELIVLLSLTRERMQDVLTRLVIASVLMIALGYPGEVSDNTGTQALFFVLAMIPFVYILRVLWKELAAEIAEE--DG 177
12 RYVDWLLTVPLLLVELIVLLSLTRERMQDVLTRLVIASVLMIALGYPGEVSDNTGTQALFFVLAMIPFVYILRVLWKELAAEIAEE--DG 177
13 RYVDWLLTVPLLLVELIVLLSLTRERMQDVLTRLVIASVLMIALGYPGEVSDNTGTQALFFVLAMIPFVYILRVLWKELAAEIAEE--DG 160
K
-CQ-AF-ALRLIVTIGWAIYPIGY-FGY-LG-AN-E-AVNIIYNYADLINK-AFGLVIWAAA--DS----------------
190 200 210 220 230 240 250 260
1 SCQKAFNALRLIVTIGWAIYPIGYVFGYLLESSNIN-AVNIIYNYADLINKTAFGLVIWAAAIKDSEASA*------------ 233
2 SCQKAFHALRLIVTVGWAIYPIGYFWGYMLDGANVE-AVNIIYNYADLINKMAFGLVIWAAAKQDSKVE*------------- 232
3 SCQKAFNALRLIVTIGWAIYPIGYVFGYLLESSNIN-AVNIIYNYADLINKTAFGLVIWAAAIKDSEASA------------- 232
4 SCQKAFHALRLIVTVGWAIYPIGYFWGYMLDGANVE-AVNIIYNYADLINKMAFGLVIWAAAKQDSKVE-------------- 231
5 SCQKAFHALRLIVTVGWAIYPIGYFWGYMLDGANVE-AVNIIYNYADLINKMAFGLVIWAAAKQDSKVE-------------- 231
6 SCQKAFHALRLIVTVGWAIYPIGYFWGYMLDGANVE-AVNIIYNYADLINKMAFGLVIWAAAKQDSKVE-------------- 231
7 ACQNAFKALRLIVTIGWAIYPIGYFWGYMLGSANMN-AVNIIYNYADLINKTAFGLVIWAAAKKDSNISV------------- 232
8 KVKELIEQTRMLLMISWLFYPVAYIFNLAGGTAEAEIGVQVGYTIADIVSKCVYGVMVYFIAREKTMLDSAPASAKAAAAKA* 260
9 KVKELIEQTRMLLMISWLFYPVAYIFNLAGGTAEAEIGVQVGYTIADIVSKCVYGVMVYFIAREKTMLDSAPASAKAAAAKA- 259
10 KVKELIEQTRMLLMISWLFYPVAYVFNLAGGTAEAEIGVQVGYTIADIVSKCVYGVMVYFIAREKTMLDSAPASAKVAAAKA- 259
11 KVKELIEQTRMLLMISWLFYPVAYIFNLAGGTAEAEIGVQVGYTIADIVSKCVYGVMVYFIAREKTMLDSAPVSAK-ATAKA- 258
12 RVKELIEQTRMLLMISWLFYPVAYVFNLAGGTAEAEIGVQVGYTIADIVSKCVYGVMVYFIAREKTMLDSAPASAKVATAKA- 259
13 KVKELIEQTRMLLMISWLFYPVAYIFNLAGGTAEAEIGVQVGYTIADIVSKCVYGVMVYFIAREKTMLDSAPVSAK-AAAKA- 241
