# Supplementary material for: Metapangenomics reveals depth-dependent shifts in metabolic potential for the ubiquitous marine bacterial SAR324 lineage
Source: Microbiome. 2021 Aug 13;9:172. doi: 10.1186/s40168-021-01119-5 (PMC8364033; doi:10.1186/s40168-021-01119-5)
Supplement: Supplementary file 14 — Additional file 13: Supplementary Figure 10. Phylogenetic reconstruction of SAR324 RuBisCO types. RuBisCO protein sequences retrieved from SAR324 population genomes (in red) were aligned on type references from Tabita et al 2007 using MUSCLE. Neighbor Joining phylogenetic reconstruction using a Poisson model; Bootstrapping of 1000. [file 40168_2021_1119_MOESM14_ESM.pdf]

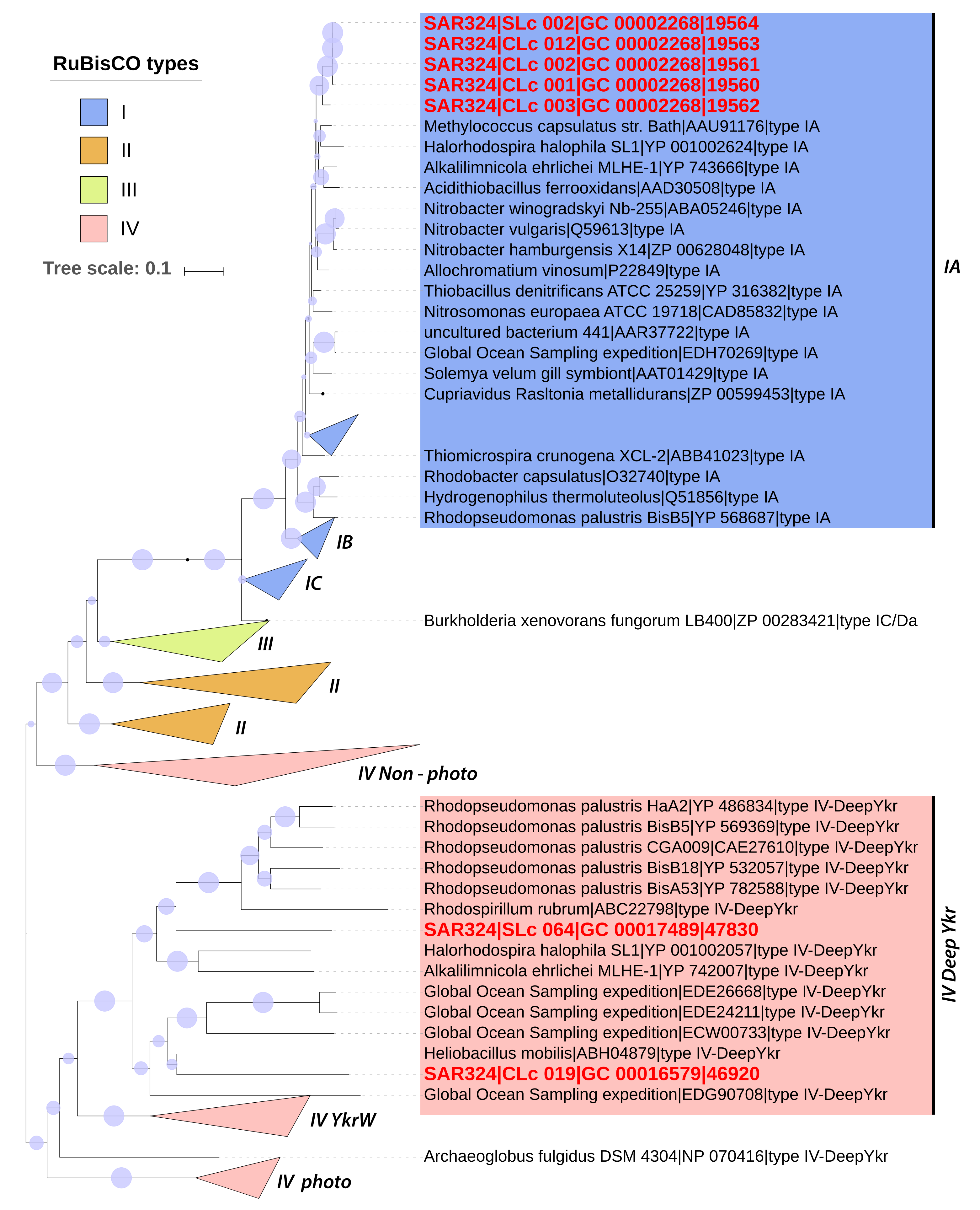
RuBisCO types
I
II
III
IV
Tree scale: 0.1
SAR324|SLc 002|GC 00002268|19564
SAR324|CLc 012|GC 00002268|19563
SAR324|CLc 002|GC 00002268|19561
SAR324|CLc 001|GC 00002268|19560
SAR324|CLc 003|GC 00002268|19562
Methylococcus capsulatus str. Bath|AAU91176|type IA
Halorhodospira halophila SL1|YP 001002624|type IA
Alkalilimnicola ehrlichei MLHE-1|YP 743666|type IA
Acidithiobacillus ferrooxidans|AAD30508|type IA
Nitrobacter winogradskyi Nb-255|ABA05246|type IA
Nitrobacter vulgaris|Q59613|type IA
Nitrobacter hamburgensis X14|ZP 00628048|type IA
Allochromatium vinosum|P22849|type IA
Thiobacillus denitrificans ATCC 25259|YP 316382|type IA
Nitrosomonas europaea ATCC 19718|CAD85832|type IA
uncultured bacterium 441|AAR37722|type IA
Global Ocean Sampling expedition|EDH70269|type IA
Solemya velum gill symbiont|AAT01429|type IA
Cupriavidus Rasltonia metallidurans|ZP 00599453|type IA
Thiomicrospira crunogena XCL-2|ABB41023|type IA
Rhodobacter capsulatus|O32740|type IA
Hydrogenophilus thermoluteolus|Q51856|type IA
Rhodopseudomonas palustris BisB5|YP 568687|type IA
IA
IB
IC
Burkholderia xenovorans fungorum LB400|ZP 00283421|type IC/Da
III
II
II
IV Non - photo
Rhodopseudomonas palustris HaA2|YP 486834|type IV-DeepYkr
Rhodopseudomonas palustris BisB5|YP 569369|type IV-DeepYkr
Rhodopseudomonas palustris CGA009|CAE27610|type IV-DeepYkr
Rhodopseudomonas palustris BisB18|YP 532057|type IV-DeepYkr
Rhodopseudomonas palustris BisA53|YP 782588|type IV-DeepYkr
Rhodospirillum rubrum|ABC22798|type IV-DeepYkr
SAR324|SLc 064|GC 00017489|47830
Halorhodospira halophila SL1|YP 001002057|type IV-DeepYkr
Alkalilimnicola ehrlichei MLHE-1|YP 742007|type IV-DeepYkr
Global Ocean Sampling expedition|EDE26668|type IV-DeepYkr
Global Ocean Sampling expedition|EDE24211|type IV-DeepYkr
Global Ocean Sampling expedition|ECW00733|type IV-DeepYkr
Heliobacillus mobilis|ABH04879|type IV-DeepYkr
SAR324|CLc 019|GC 00016579|46920
Global Ocean Sampling expedition|EDG90708|type IV-DeepYkr
IV Deep Ykr
IV YkrW
Archaeoglobus fulgidus DSM 4304|NP 070416|type IV-DeepYkr
IV photo
